# **β**-Catenin–mediated immune evasion pathway frequently operates in primary cutaneous melanomas

**DOI:** 10.1172/JCI95351

**Published:** 2018-04-16

**Authors:** Jérémie Nsengimana, Jon Laye, Anastasia Filia, Sally O’Shea, Sathya Muralidhar, Joanna Poźniak, Alastair Droop, May Chan, Christy Walker, Louise Parkinson, Joanne Gascoyne, Tracey Mell, Minttu Polso, Rosalyn Jewell, Juliette Randerson-Moor, Graham P. Cook, D. Timothy Bishop, Julia Newton-Bishop

**Affiliations:** 1 Leeds Institute of Cancer and Pathology, University of Leeds, Leeds, United Kingdom.; 2 National Heart and Lung Institute, Imperial College, London, United Kingdom.; 3 Medical Research Council (MRC) Medical Bioinformatics Centre, University of Leeds, Leeds, United Kingdom.; 4 Yorkshire Regional Genetics Service, Leeds Teaching Hospitals NHS Trust, Leeds, United Kingdom.

**Keywords:** Immunology, Oncology, Cancer immunotherapy, Expression profiling, Melanoma

## Abstract

Immunotherapy prolongs survival in only a subset of melanoma patients, highlighting the need to better understand the driver tumor microenvironment. We conducted bioinformatic analyses of 703 transcriptomes to probe the immune landscape of primary cutaneous melanomas in a population-ascertained cohort. We identified and validated 6 immunologically distinct subgroups, with the largest having the lowest immune scores and the poorest survival. This poor-prognosis subgroup exhibited expression profiles consistent with β-catenin–mediated failure to recruit CD141^+^ DCs. A second subgroup displayed an equally bad prognosis when histopathological factors were adjusted for, while 4 others maintained comparable survival profiles. The 6 subgroups were replicated in The Cancer Genome Atlas (TCGA) melanomas, where β-catenin signaling was also associated with low immune scores predominantly related to hypomethylation. The survival benefit of high immune scores was strongest in patients with double-WT tumors for *BRAF* and *NRAS*, less strong in *BRAF*-V600 mutants, and absent in *NRAS* (codons 12, 13, 61) mutants. In summary, we report evidence for a β-catenin–mediated immune evasion in 42% of melanoma primaries overall and in 73% of those with the worst outcome. We further report evidence for an interaction between oncogenic mutations and host response to melanoma, suggesting that patient stratification will improve immunotherapeutic outcomes.

## Introduction

Melanoma is an immunogenic tumor, and the presence of histopathologically detected tumor-infiltrating lymphocytes (TILs) is associated with increased patient survival and reduced risk of metastasis (1). For stage IV melanoma, patient outcomes can be improved through the use of immunotherapy. PD-1 blockade is the best-tolerated and most effective drug treatment so far (2), but it only benefits approximately 35% of patients (3–5). This, coupled with the potential for severe side effects, highlights the need to identify those patients whose outcomes will be improved using alternative regimes (6). In an attempt to better understand the drivers of antimelanoma immune responses, we have characterized the transcriptomes from a large collection of primary tumors in a treatment-naive population. It is our hypothesis that the identification of these subgroups will be of benefit in predicting responses to immunotherapy and other targeted therapies.

Predictive biomarkers using archival tumor tissue are desirable, but degraded RNA/DNA has been identified as one of the major challenges to genomic studies (7). Transcriptomic tumor profiling and bioinformatic imputation have recently allowed the categorization of the immune component (immunome) of tumors (8, 9), in which the presence of different proportions of tumor-infiltrating immune cells is inferred from the overrepresentation of transcripts specific to particular immune cell types. Here, we have used a modification of this technique to characterize the heterogeneous immune landscape in 703 primary cutaneous melanomas from a population-ascertained cohort (the Leeds Melanoma Cohort [LMC]) (10, 11) and examined whether the inferred immune subgroups reflect patient survival characteristics. We have coupled this with an analysis of candidate immune evasion pathways and correlations with driver oncogene mutations and tested our findings in a second data set from The Cancer Genome Atlas (TCGA), which includes primarily metastatic tumors (*n* = 369) with a subset of primaries (*n* = 103). Our results highlight substantial heterogeneity in the immune landscape across melanoma primaries and metastases and show evidence for immune inhibition by β-catenin signaling in a significant proportion of tumors.

## Results

Whole transcriptomes were derived from 703 formalin-fixed paraffin wax–embedded (FFPE) primary cutaneous melanomas from the LMC (median follow-up, 7.5 years), using the Illumina DASL HT12.4 array. These data were normalized, then randomly divided into training (two-thirds) and test sets (one-third) (see Methods, Supplemental Table 1, and Supplemental Figure 1; supplemental material available online with this article; https://doi.org/10.1172/JCI95351DS1). The definition of the immunome originally developed for application to colorectal cancer (8) was adapted to melanoma by the filtering out of genes expressed by melanoma and melanocyte cell lines, resulting in a set of 380 genes specific to 24 immune cell types, referred to herein as the “Melanoma Immunome” (see Methods). The gene list, inferred immune cells, immunity type (innate or adaptive), and relationship between some of these cells as reported by Bindea et al. (8) are summarized in Supplemental Table 2 and Supplemental Figure 2.

### Consensus cluster analysis.

**Figure 1:**
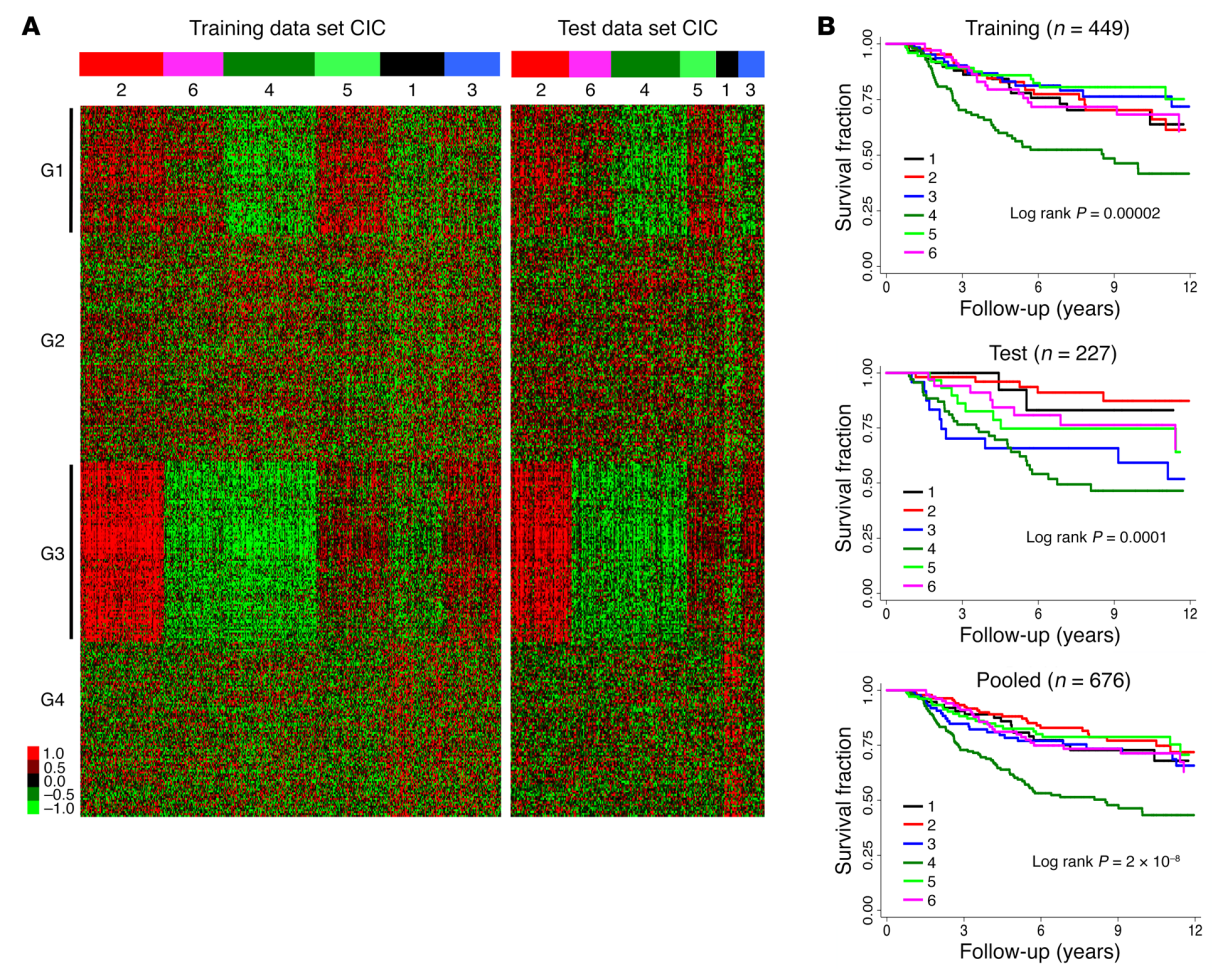
Tumor classification. (**A**) Consensus immunome clusters (CICs) in the LMC training (*n* = 465) and test (*n* = 238) data sets ordered according to the dendrogram output from ConsensusClusterPlus (see Supplemental Figure 3). The details of gene clusters G1–G4 are given in Supplemental Figure 4. (**B**) Differential melanoma-specific survival for patients with tumors in the 6 CICs in the training, test, and pooled data sets unadjusted for histological factors. Cluster size: 11%, 21%, 13%, 25%, 15%, and 15% for CICs 1–6, respectively. Cause of death was unknown or was not melanoma for 27 patients, and they were excluded from survival analysis.

Using primary tumor transcriptomes, we conducted consensus cluster analysis (12, 13) of the training sample set (see Methods and Supplemental Figure 3). This revealed 6 distinct subgroups reproducible in the test data set and referred to herein as consensus immunome clusters (CICs) (Figure 1A). Examination of the molecular characteristics of these CICs (see below) allowed us to further characterize them as high immune (CIC2), low immune/keratin rich (CIC6), low immune/β-catenin high (CIC4), intermediate immune/keratin rich (CIC5), low immune/β-catenin low (CIC1), and intermediate immune/keratin poor (CIC3). Four groups of genes (Figure 1A; see Methods for more details) defined these clusters, 2 groups of which appeared as the most discriminant (G1 and G3; Figure 1A). Each gene cluster was enriched in expression of genes attributed to particular immune cell subtypes (Supplemental Figure 4). G1 was strongly enriched in genes characteristic of innate immune cells such as macrophages (*P* = 0.001), mast cells (*P* = 10^–8^), and immature DCs (*P* = 2 × 10^–5^); G3 was strongly enriched in genes associated with adaptive immunity (cytotoxic cells, *P* = 4 × 10^–5^, and T cells, *P* = 2 × 10^–8^), and G4 was enriched in genes attributed to natural killer (NK) CD56^bright^ cells (*P* = 2 × 10^–4^). These *P* values (derived from Fisher’s exact test) were all, except for macrophages in G1, below 5.2 × 10^–4^, which corresponds to 0.05 corrected for multiple testing using the conservative Bonferroni method (24 cell types × 4 gene clusters = 96 tests). In addition to adaptive immunity, G3 had a nominal overrepresentation of genes attributed to 2 innate immune cells, activated DCs (aDCs; *P* = 0.05) and NK CD56^dim^ cells (*P* = 0.007), although this overrepresentation did not hold up upon multiple-testing adjustment. G2 had nominally significant overrepresentation of genes attributed to CD8^+^ T cells (*P* = 0.005), central memory T cells (*P* = 0.02), Th2 cells (*P* = 0.05), and eosinophils (*P* = 0.03) (Figure 1A and Supplemental Figure 4).

High immune (CIC2) and intermediate immune/keratin poor (CIC3) notably overlapped with “high immune” phenotypes inferred using signatures reported by Jonsson et al. and TCGA (14–16), and low immune/β-catenin high (CIC4) overlapped with “pigmentation,” “proliferative,” and “MITF.low,” while intermediate immune/keratin rich (CIC5) and low immune/keratin rich (CIC6) overlapped with “normal-like” and “keratin” phenotypes (Supplemental Figure 4).

Tumors classified as high immune (CIC2) were also those most frequently classified as having “brisk” TILs (34%) by the dermatopathologists who generated the biopsy reports (Table 1), while those tumors classified as low immune/β-catenin high (CIC4) presented with far fewer pathologist-reported brisk TILs (7%; Table 1), and this was associated with significantly worsened melanoma-specific survival (training set: *P* = 2 × 10^–5^; test set: 10^–4^; pooled data, *P* = 2 × 10^–8^; Figure 1B). The presence of TILs in the whole tumor and the cored area of tumor, as graded blindly by a single observer (S. O’Shea) (see Supplemental Figure 5 for examples of TIL grading), showed a similar relationship with CICs (Supplemental Table 3). The hazard ratio (HR) for melanoma death was 1.7 (*P* = 0.004) for low immune/β-catenin high (CIC4) versus the remaining CICs after adjustment for 7 other prognostic factors (Table 2). The results were maintained when the American Joint Committee on Cancer (AJCC) stage was replaced by ulceration and Breslow thickness in multivariable analyses (Supplemental Table 4). The prognostic effect of CICs was much stronger than that of *CD8A* or *CD8B* expression alone (Supplemental Table 4).

**Figure 2:**
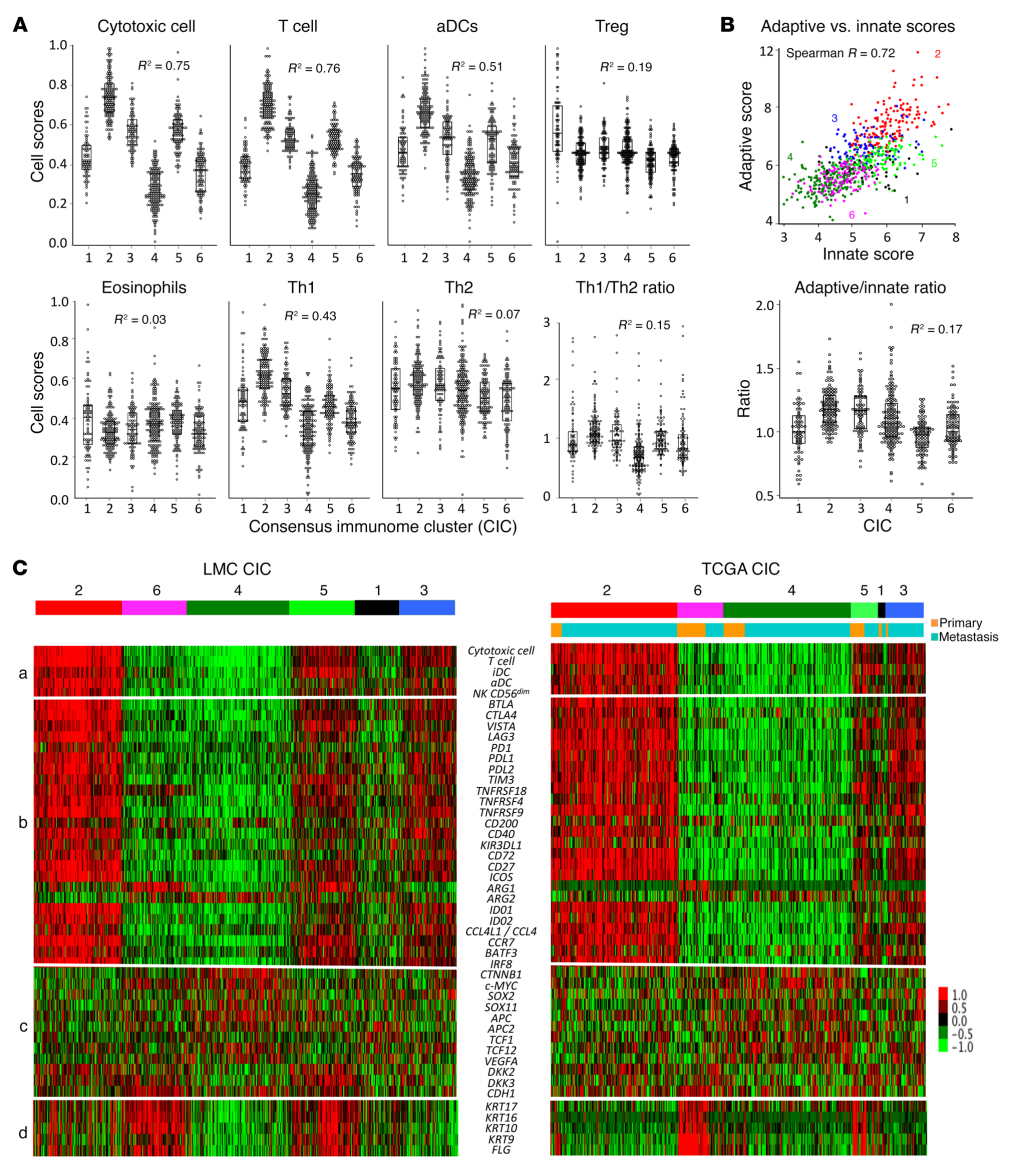
Association of immune scores with evasion mechanisms. (**A**) Distribution of selected immune cell scores in 6 CICs (pooled training and test LMC, *n* = 703). (**B**) Correlation and ratio between adaptive and innate immune scores (LMC). Owing to the high correlation between adaptive and innate scores, their ratios show a little variation between CICs. In **A** and **B**, *R*^2^ is the proportion of variance explained by the 6 CICs computed in ANOVA. Dot plots are shown alongside box plots showing the median and the interquartile range. (**C**) Correlations in LMC and TCGA (*n* = 472) between (a) 5 cell scores; (b) checkpoint and other regulatory genes; (c) β-catenin signaling genes; and (d) keratin and filaggrin expression. Based on the immune genes and keratin expression, the 6 CICs were described as low immune/β-catenin low (CIC1), high immune (CIC2), intermediate immune/keratin poor (CIC3), low immune/β-catenin high (CIC4), intermediate immune/keratin rich (CIC5), and low immune/keratin rich (CIC6).

Despite similar survival profiles, the tumors represented within CICs 1, 2, 3, and 5 and 6 had heterogeneous immune signatures (Figure 1A) and clinicopathological features. High immune (CIC2) was characterized by higher expression of immune signaling genes (in particular, cytotoxic cell genes) compared with intermediate immune/keratin poor (CIC3) and intermediate immune/keratin rich (CIC5), and yet this appeared to bestow no survival advantage (Figure 1B). Low immune/β-catenin low (CIC1) was the smallest cluster and appeared to be defined by genes from NK CD56^bright^ cells and higher Treg scores (Figure 2A). CICs 1, 2, 3, 5, and 6 differed in median age at diagnosis, mitotic rate, Breslow thickness, gender, ulceration, and AJCC stage (Table 1). For example, only 15.7% of tumors were diagnosed at AJCC stage I in intermediate immune/keratin poor CIC3, while this stage represents about 50% of intermediate immune/keratin rich (CIC5). Further analysis revealed that low immune/keratin rich (CIC6) actually had a poor outcome, comparable to that of the low immune/β-catenin high cluster (CIC4) (Table 3) when taking account of clinical factors, indicating that CIC6’s apparent good prognosis as seen in Figure 1A was driven by beneficial clinical factors. Particularly, despite very different prognoses, intermediate immune/keratin poor (CIC3) (good survival) and low immune/β-catenin high (CIC4) (poor survival) were both associated with clinicopathological features well known to be associated with poor prognosis. Significantly, the key differences between the 2 clusters were the presence of histopathologically reported TILs (Table 1) and a transcriptomic strong immune profile in intermediate immune/keratin poor CIC3 (Figure 1A).

We had 9 paired transcriptomes from primaries and metastases: 6 nodal and 3 soft tissue. CICs of these pairs are reported in Supplemental Table 5. Generally speaking, the immune status of the metastases was poorer than that of the primary tumor (participants 1, 4, and 5) or equally poor (participants 7 and 9). In 3 nodal metastases the data suggest a possible contamination of the tumor sample by nodal lymphocytes, and a keratin signature was observed in a cutaneous metastasis.

### Immune cell scores.

We generated immune cell scores by averaging gene expression data attributed to each cell type (Supplemental Table 2) and used them to further compare CICs and explore immunomodulation. These scores varied strongly across CICs (Figure 2A), but the relative score differences were consistent across the immune cell types. High immune CIC2 consistently demonstrated the highest and low immune/β-catenin high CIC4 demonstrated the lowest scores for most immune cell types. The scores for Th2 cells, Tregs, and eosinophils exhibited the lowest variation between CICs. Although there was strong statistical evidence of an orchestrated immune response across clusters, examination of the dot and box plots in Figure 2A shows a relative excess of Tregs in low immune/β-catenin low (CIC1), and although Th2 did not vary much between the CICs, the Th1/Th2 ratio was lowest in low immune/β-catenin high CIC4 in conjunction with the lowest immune score. Consistent with the results from consensus classification (Figure 1A), the correlation between innate and adaptive immune scores was weaker within intermediate immune/keratin poor CIC3 and intermediate immune/keratin rich CIC5 compared with the rest (Figure 2B).

Genes encoding PD-1, PDL-1, PDL-2, CTLA4, TIM-3, LAG3, VISTA, BTLA, IDO1, IDO2, ICOS, CD27, and CD72 had their highest expression in the tumors with the strongest T cell infiltration (Figure 2C), while *CD200* had no clear association with CICs. Immunostimulatory transcription factors encoded by *IRF8* and *BATF3* were also highly correlated with both T cell and aDC scores, as was expression of *CCR7* and *CCL4L1* (Figure 2C).

The expression of *CTNNB1* (encoding β-catenin) and its downstream targets such as *TCF12*, *APC2*, *SOX2*, *SOX11*, and *MYC* was inversely correlated with T cell cytotoxicity scores (Figure 2C) with up to 1.8-fold higher levels in CIC4 compared with CIC2 (Supplemental Table 6). A score for the β-catenin signaling pathway based on these genes was significantly different between CICs and was strongly negatively correlated with the T cell score (Spearman *R* = –0.43, compared with *R* = –0.31 between T cell score and *CTNNB1* expression). In contrast, the genes coding for inhibitors of this pathway such as *DKK2* and *DKK3* were upregulated in the good-prognosis groups high immune (CIC2) and intermediate immune/keratin rich (CIC5). E-cadherin plays a role in posttranscriptional regulation of β-catenin, and we postulated it might reduce the immunoinhibitory effect of β-catenin; we found its expression weakly but positively correlated with that of β-catenin (Figure 2C; Spearman *R* = 0.15). We found no evidence that the ratio between E-cadherin and β-catenin expression more strongly correlated with T cell infiltration (data not shown). We used X-tile (17) to identify cut points in *CTNNB1* expression best predicting reduction in T cell score, and from this analysis we calculated that 59% of immune low/β-catenin high CIC4 tumors exceeded this level compared with only 30% of the total population-based cohort (Fisher’s exact *P* = 6 × 10^–21^). A similar analysis based on β-catenin pathway score indicated that 42% of the cohort have upregulation of the pathway, but this figure was 73% in CIC4 (Fisher’s exact *P* = 3 × 10^–21^).

### Replication.

To validate our observations, we used the TCGA data set. The 6 CICs derived from the LMC were also visible in this independent data set and were found in both primaries and metastases. As in the LMC, CICs 2 and 4 were the largest, with 160 tumors each, comprising both primaries and metastases (Table 4). CICs 6, 5, and 1 contained the largest proportion of primary tumors (61%, 50%, and 44%, respectively), although low immune/β-catenin low (CIC1) was very small, with only 9 tumors in total (Table 4). Similarly to the LMC, TCGA tumors classified within low immune/β-catenin high CIC4 were associated with the poorest survival (HR = 1.44, *P* = 0.01 for CIC4 vs. all others after adjustment for sex, age, and tumor type; see Table 5). All the observations made in the LMC regarding checkpoint molecules, immunosuppressive enzymes, and immunostimulatory transcription factors were recapitulated in both primaries and metastases of the TCGA data set, i.e., the signatures for all these variables were most highly expressed in high immune CIC2 and repressed in low immune/β-catenin high CIC4 (Figure 2C). Similarly, the expression levels of the β-catenin pathway genes *CTNNB1*, *TCF12*, *APC2*, *SOX2*, *SOX11*, and *MYC* were all inversely correlated with immune scores in the TCGA data set, while their highest expression was also observed in low immune/β-catenin high CIC4 (Supplemental Table 6). In a sensitivity analysis, all LMC and TCGA primaries were pooled into 1 data set, and a new consensus clustering was conducted. The 6 tumor clusters and 4 gene clusters were again found (Supplemental Figure 7), indicating robustness of the approach used.

**Figure 3:**
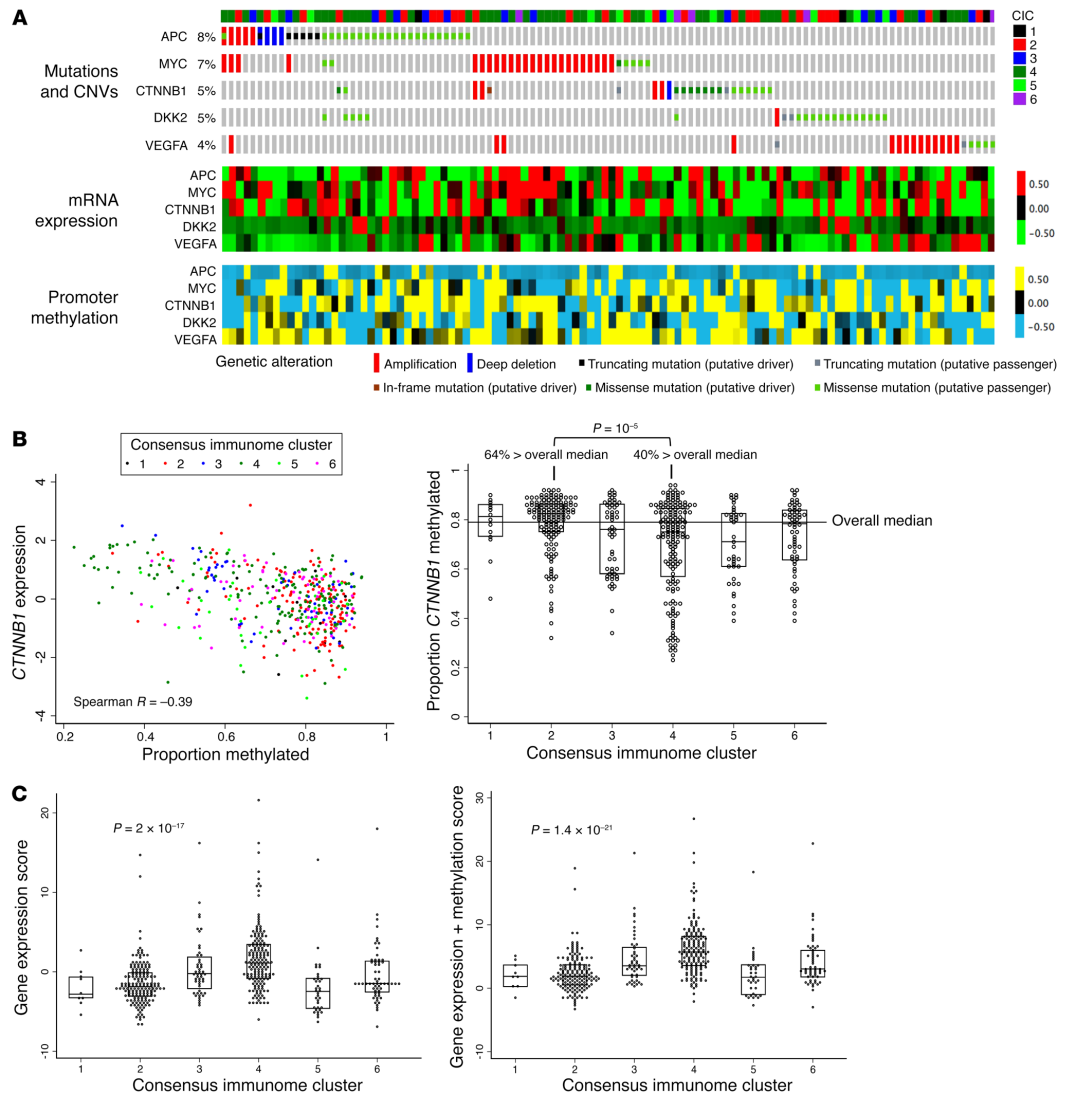
β-Catenin pathway regulation by mutations, copy number variation, and promoter methylation. (**A**) Plot of mutations, copy number variations (CNVs), mRNA expression, and promoter methylation in genes representing β-catenin pathway in TCGA data set. Figure shows the tumors with at least 1 gene mutated or altered (*n* = 109) and genes affected by those changes in at least 4% of tumors. The samples (columns) are ordered from the most altered to the least altered. The top annotation bar represents 6 CICs. Fifty-five samples had at least 1 mutation, 44 at least 1 CNV; 5 had both. (**B**) Correlation between expression of *CTNNB1* and its promoter methylation and statistical difference between CICs 2 and 4 (Mann-Whitney test). (**C**) A score combining expressions of 9 β-catenin signaling genes and their methylation has a higher correlation with the 6 CICs than a score from expression data alone (Kruskal-Wallis). In **B** and **C**, the median and the interquartile range are shown in dot and box plots (*n* = 472).

We additionally used melanoma TCGA methylation (*n* = 472), mutation (*n* = 287), and copy number variation (*n* = 367) data to examine the contribution of epigenetic and structural changes to the transcriptomic characteristics of CIC4. Overall, several genes in the β-catenin signaling pathway were amplified and/or had mutations in a proportion of tumors (8% for *APC*, 7% for *MYC*, and 5% for *CTNNB1*), although the majority were putative passenger mutations as shown in Figure 3A. There were, however, more amplifications (19/133) in low immune/β-catenin high CIC4 compared with the rest of the samples (12/235), with *P* = 0.002. Deletions (*n* = 5) and driver mutations (*n* = 15) were rarer and showed no association with clusters. It is noticeable from the OncoPrint graph (Figure 3A) that the majority of the alterations in the analyzed genes did not co-occur. As expected, expression of *CTNNB1* itself and genes in the β-catenin signaling pathway was strongly negatively correlated with promoter methylation across all CICs (Spearman *R* = –0.58 to –0.22). Promoter methylation of β-catenin (*CTNNB1*) was markedly lower in low immune/β-catenin high CIC4 compared with other clusters, in particular compared with high immune CIC2 (*P* = 10^–5^; Figure 3B). A score combining expressions of 9 β-catenin signaling genes was associated with the tumor clusters (*P* = 2 × 10^–17^), and when methylation of these genes was further added, the association was stronger (*P* = 1.4 × 10^–21^) (Figure 3C). The contribution from putative activating mutations and copy number changes to the pathway score was comparatively weaker (Supplemental Figure 6).

### Immune scores and driver mutation.

**Figure 4:**
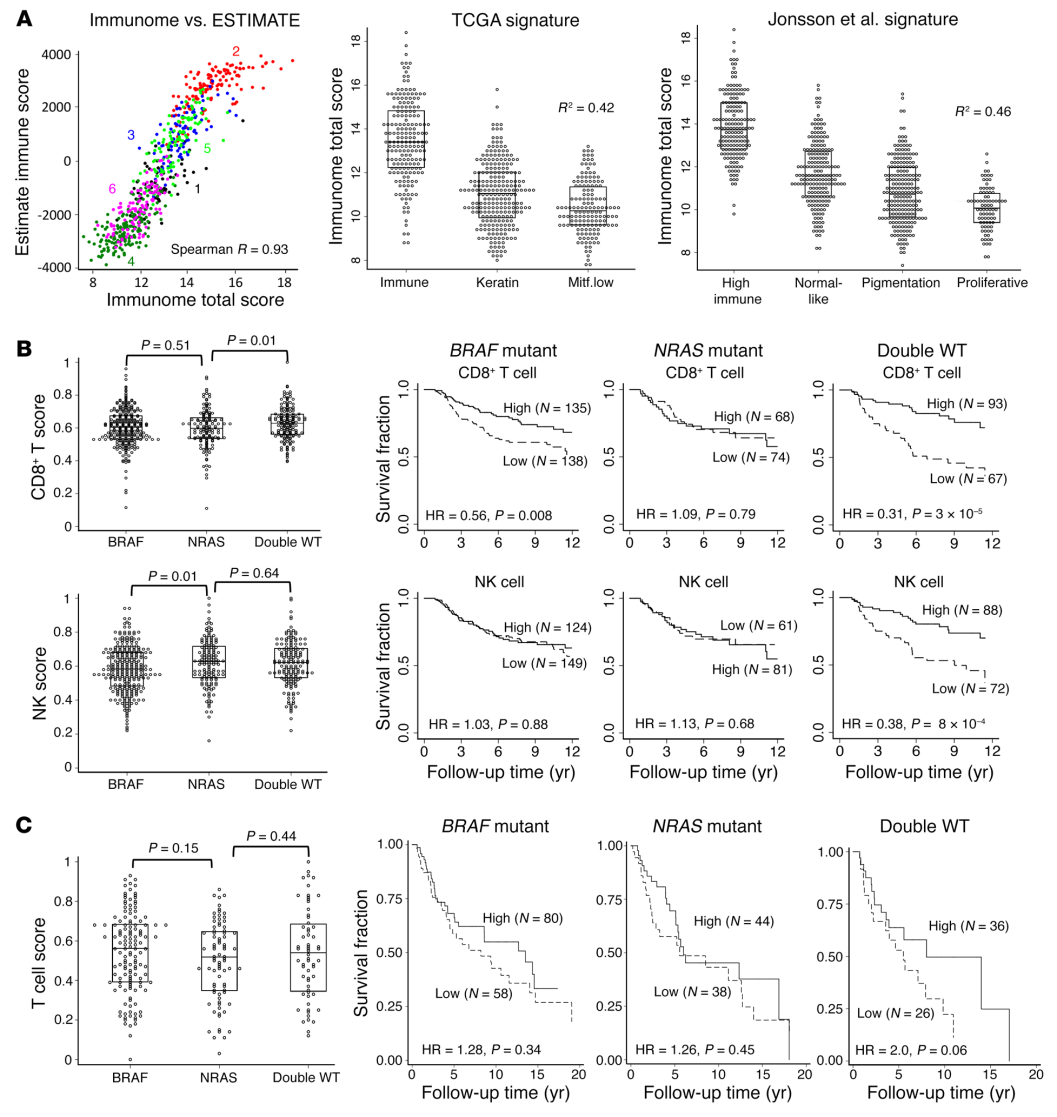
Immune score interaction with driver mutation. (**A**) Correlation between immunome total score in the LMC (*n* = 703) and its equivalent from ESTIMATE (18) and comparison with 2 published molecular signatures (14, 15). (**B**) Distribution of CD8^+^ T cell and NK cell scores and their association with melanoma-specific survival by driver mutation in LMC. (**C**) Association between T cell score and overall survival by driver mutation in TCGA data set (*n* = 287). In **B** and **C**, the difference in immune cell scores (dot and box plots with median and interquartile range) does not explain the difference in survival. Cox proportional hazards model used in survival analyses; Kruskal-Wallis used to test score distribution by mutation status.

By combining scores of 24 cell types of the immunome, we generated a measure of the total immune component of each tumor; these immune scores demonstrated good correlation with an overall immune score generated from the same data set using another scoring algorithm (Estimation of Stromal and Immune Cells in Malignant Tumors Using Expression Data [ESTIMATE]; ref. 18 and Figure 4A). When we applied published melanoma molecular signatures (14, 15) to our data, we observed that tumors with the highest immune scores were also more likely to fall into molecular profiles previously characterized as high immune (Figure 4A). Multiple immune scores, including T cell, CD8^+^ T cell, cytotoxic cell, Th1, aDC, B cell, and total immune score, were highly prognostic in the LMC and were closely replicated in TCGA (Table 6). We sought to assess whether the most prevalent oncogenic mutations in *BRAF* (V600) and *NRAS* (codons 12, 13, and 61) may also moderate immune responses. In the LMC, CD8^+^ T cell and NK cell scores were strongly predictive of melanoma-specific survival in double-WT tumors, but less so in *BRAF*-mutated and not at all in *NRAS*-mutated tumors (Figure 4B); these differences were not matched by differences in cell scores (Figure 1A). We observed no difference in pathologist-reported TILs between *NRAS*-mutated and WT tumors despite previous observations that *NRAS*-mutated melanoma primaries are less likely to have brisk TILs (19). Neither did we see evidence of higher *CD274* (encoding *PDL-1*) in *NRAS*-mutated tumors (20). The differential prognostic effect of CD8^+^ T and NK cell scores by driver mutation persisted when we adjusted for age, sex, site, and AJCC staging. Importantly, this observation was again confirmed in the metastatic tumors of TCGA (*n* = 287), with the T cell score showing a similar distribution in *BRAF*-mutated, *NRAS-*mutated, and double-WTs but showing no prognostic effect in *NRAS*-mutated tumors (Figure 4C).

### Protein data.

**Figure 5:**
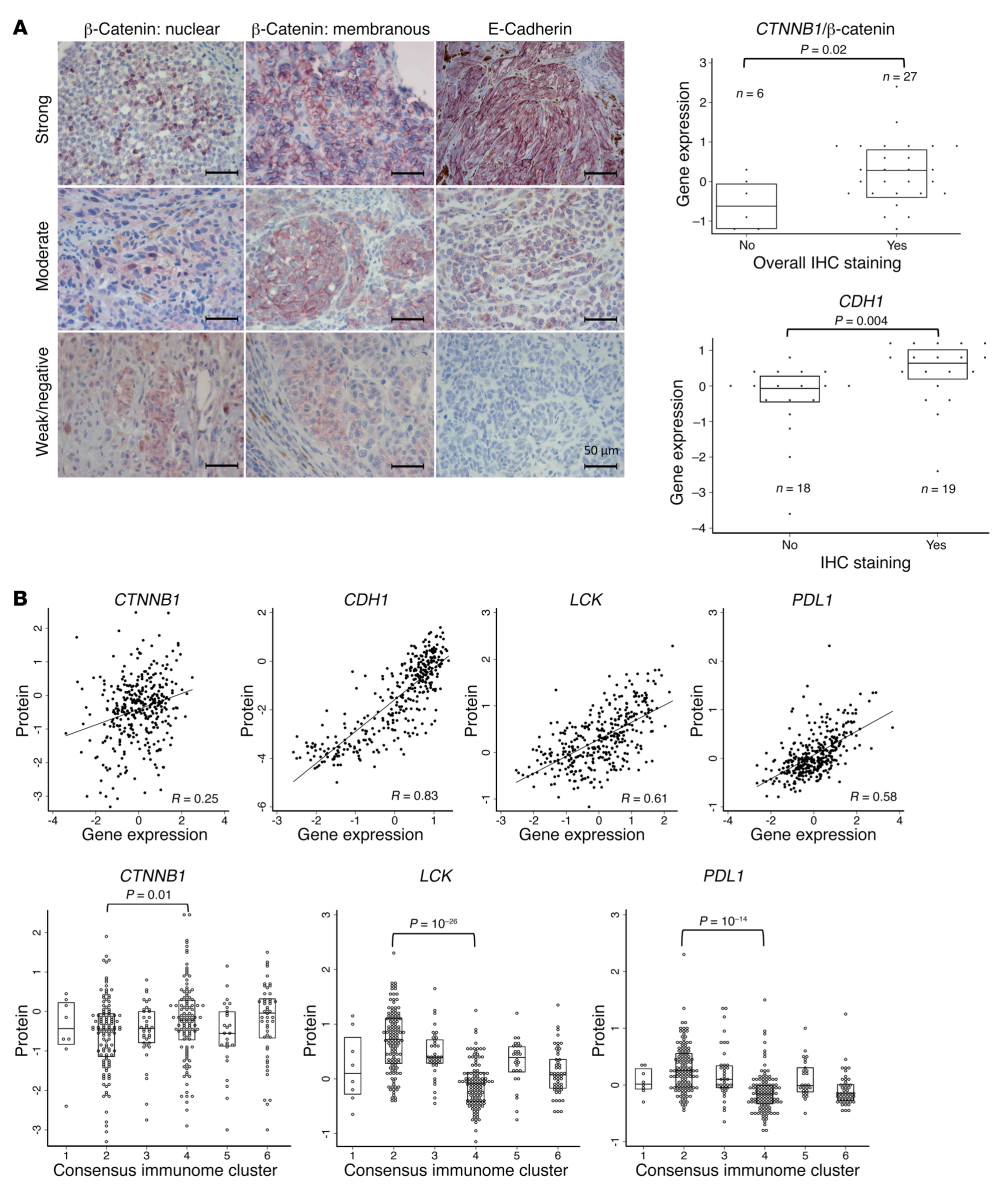
Validation with IHC staining and protein data. (**A**) Representative images of IHC staining in the LMC demonstrating strong, moderate, and weak/negative expression of β-catenin localized to the nucleus (left column) and to the membrane (middle column) and expression of E-cadherin (right column) (CTNNB1 and CDH1 were stained in 33 and 37 tumors, respectively). Dot and box plots (median and interquartile range) show mRNA expression of *CTNNB1* and *CDH1* compared with IHC staining. (**B**) Protein (RPPA) data for LCK, PDL-1, CTNNB1, and CDH1 in TCGA skin melanomas (*n* = 354) in comparison with mRNA expression and CICs. Kruskal-Wallis test and Spearman correlation were used. Box plots show the median and the interquartile range.

In the next validation step, we conducted immunohistochemical (IHC) staining of β-catenin and its membranous ligand E-cadherin in the cell membrane and of E-cadherin in a subset of the LMC data set (33 and 37 tumors, respectively). Figure 5A shows examples of tumors with “low” and “high” staining and the overall concordance between reported gene expression and the degree of IHC staining. Both β-catenin and E-cadherin were expressed only in tumor cells rather than stromal cells; E-cadherin was strongly expressed by tumor cells and epidermal cell membranes. There was a good agreement between gene expression and IHC staining of the protein for both genes, with *P* = 0.02, 0.02, and 0.06 for overall, cytoplasmic, and membranous β-catenin, respectively, and *P* = 0.004 for E-cadherin. We also downloaded the Reverse Phase Protein Array (RPPA) data from The Cancer Proteome Atlas for 354 of the 472 TCGA skin melanoma samples and compared these protein data with their corresponding gene expression for β-catenin, E-cadherin, LCK, and PDL-1. RPPA measures protein levels on a continuous scale, and Figure 5B shows that those values correlate well with mRNA expression (*R* = 0.25–0.83). As we observed in the gene expression data, low immune/β-catenin high CIC4 had a significantly higher protein expression of CTNNB1 (*P* = 0.02) and a lower expression of LCK (*P* = 10^–26^) and CD274 (PDL-1) (*P* = 10^–14^) compared with high immune CIC2 (Figure 5B). Taken together, these IHC and RPPA data demonstrate concordance between gene expression and protein levels for these genes.

## Discussion

This study has considerable strengths as it is derived from population-ascertained melanoma patients, enabling more confident generalization of conclusions. The data set is one of the largest internationally and is unique in that it is derived from primary tumors. The sampling method allowed identification of transcriptomic signals from the tumor environment, which was our intent, as tumor/host interaction is crucial in determining outcomes from this and other cancers. The sampling was carefully done, however, in order to compare one tumor with another. One weakness of the study is that there is some bias of sampling in that very thin primaries were less likely to be sampled, although this is much less of a problem than in studies using fresh frozen samples. A second weakness is that using a tissue microarray (TMA) core may result in sampling of hair follicles with a recognizable site-specific signature such as some tumors classified in CIC5 and CIC6 (high keratin and filaggrin expression). The identification of similar keratin signatures in Jonsson et al. (14) and TCGA (15) suggests, however, that this is an issue intrinsic to transcriptomic studies of this type irrespective of sampling method. The resulting data, we argue, are complementary to smaller-scale studies in which tumors are disaggregated and then flow-cytometrically sorted in terms of understanding the biology of interactions between host and tumor.

A significant outcome of the study is that a single 0.6-mm diameter of FFPE tumor core was sufficient to generate array-based transcriptomic data in 96.4% of tumors sampled; the quality of the data is evidenced by the strong correlation between inferred immune cell scores and the presence of brisk TILs reported by specialist histopathologists and by a single observer in our research group. Moreover, we see very similar results from clusters identified in formalin-fixed primaries versus clusters identified in TCGA data, in which gene expressions were generated using RNA-Seq from fresh frozen tumor. In clinical biomarker development terms, we anticipate that sampling of all but a small proportion of very thin AJCC stage I tumors would be feasible from FFPE tumor (especially as arrays requiring very small amounts of RNA are now in use) and therefore that transcriptomic studies designed to identify predictive biomarkers are now possible for tumor blocks stored from mature clinical trials. As the generation of sufficient RNA is possible now from single 5-μm slides, greater control of sampling should be possible. Finally, the most significant outcome, if we interpret our findings in the context of a general population of melanoma cases, was that CIC4, the worst-prognosis group, which is the most prevalent of the 6 clusters, represents, we estimate, some 36% of stage III and 30% of stage II melanoma but only 12% of stage I melanoma.

Previous studies have shown an important prognostic role played by immune activation in metastatic disease (21) and evidence that the identification of immune signatures may have predictive value for checkpoint blockade (22). Our study shows that immune signatures also predict survival in primary melanoma, offering hope that efficient molecular predictive biomarkers can be derived for adjuvant immunotherapies. This is of relevance as survival benefit from ipilimumab as melanoma adjuvant therapy has been reported and the drug approved for adjuvant use in the United States. Recent comparison of nivolumab and ipilimumab as adjuvant therapies for resected stage III and IV melanoma has also been reported (23, 24). We provide a detailed description of the immune landscape in tumors corroborated by histological evidence of TILs and inferences from other published tools (14, 15, 18) that we aim to develop and test in adjuvant clinical trials in order to identify predictive biomarkers.

The primary tumor clusters defined by immune profiles were strongly associated with histological characteristics such as Breslow thickness, mitotic rate, and ulceration, yet showed an independent prognostic value. As in previous reports (8, 21), we found that the tumors associated with the worst survival had gene expression patterns indicative of little cytotoxic T cell infiltration. We further found evidence specifically of fewer DCs, macrophages, and mast cells (Figure 1A and Supplemental Figure 4). There are few published data relating to the role of mast cells in melanoma survival, but a small histochemical study recently reported that lower mast cell numbers were associated with melanoma progression (25). Arginase 1 and mast cell genes were upregulated in low immune/keratin rich (CIC6) and intermediate immune/keratin rich (CIC5), which were on average thinner and had predominantly “normal-like” and “keratin” phenotypes using previously described signatures (refs. 14–16 and Supplemental Figure 4). Since mast cells more commonly occur in the epidermis as does arginase 1 expression (Human Tissue Atlas; https://www.proteinatlas.org/), concern exists that this signature may reflect possible sampling of healthy skin.

Activation of T cells by DCs is required to promote antitumor immunity, and this depends on the presence of appropriate transcription factors and cytokines (26). Our study demonstrates that expression of genes such as those coding for chemokine and chemokine receptors can be detected in archived primary tumors at the transcript level, offering a viable alternative to flow cytometry or cytokine assays in biomarker studies, given the practical limitation of these assays in clinical settings (27).

By attributing a score to innate and adaptive immunity as previously defined (8), we were able to assess whether the immune cells characterizing each CIC were suggestive of a polarized response (Figure 2B). Although the innate and adaptive immune scores were very highly correlated, they were less so in intermediate immune/keratin poor CIC3 (more adaptive) and intermediate immune/keratin rich CIC5 (more innate), but the lack of survival difference in these 2 groups may indicate that any type of immune response is better than none, as has been implied in studies of outcomes from different immunotherapeutic regimes (28, 29). Moreover, the lack of survival differences between CICs 1, 2, 3, and 5 may also reflect the differences in prognostic histopathological features. As an example, multivariable analysis indicated that the good survival in low immune/keratin rich (CIC6) was in part determined by these factors (Tables 1 and 3). The most informative comparison is between intermediate immune/keratin poor CIC3 and low immune/β-catenin high CIC4, as they have similar histopathological features (e.g., 52% and 49% ulcerated tumors, respectively, the highest mitotic rate of all clusters, and median Breslow thickness of 2.7 and 3.2 mm, respectively; Table 1). The melanoma-specific survival was significantly worse in low immune/β-catenin high CIC4, however, and this was associated with lower immune scores and less histopathologist-reported brisk TILs.

The total immune score data were consistent with published literature (14, 18). Several individual immune cell scores showed a significant association with patient survival in univariable analysis, including T cells, cytotoxic cells, CD8^+^ T cells, B cells, follicular helper T (Tfh) cells, Th17 cells, and Th1 cells, which is consistent with existing literature (21, 30, 31). However, the high correlation between the scores for all immune cells as seen in Figure 1A makes it difficult to disentangle the role played by each of them. Although not significant after multiple testing and not replicated in TCGA, Th2 cell and eosinophil scores, and eosinophils were the only cell types with a nominally detrimental effect on survival (Table 6). Th2-mediated inflammation acting to promote tumors has been reported (32), and although its score had little variation across the CICs in our data, the Th1/Th2 ratio did vary and was lower in the poor prognostic cluster low immune/β-catenin high. Drawing the data together, low immune/β-catenin high CIC4 had the lowest immune scores overall, the worst prognosis, increased β-catenin signaling, increased levels of arginase 2, and lower Th1/Th2 ratio, all consistent with a protumorigenic immunosuppressive microenvironment.

Treg scores showed little variation across the CICs, and we cannot exclude the possibility that this was due to the intrinsic limitation of using gene expression to infer immune cells (Tregs were represented by a single gene, *FOXP3*). There was some evidence of higher Tregs in low immune/β-catenin low (CIC1), which had a low immune overall score but high CD56^bright^ NK cells and good survival. This cluster is an unusual tumor group, as it was also associated with 52% tumors on the limbs (a good prognostic factor for melanoma), high *BRAF* driver mutation status (55%), yet a high frequency of tumor ulceration (38%; Table 1). The significance of this group is as yet unclear.

The survival of low immune/β-catenin high CIC4 participants was worst, with low immune-keratin rich (CIC6) similarly poor when data were adjusted for histopathological features. The survival of the additional immune groups was, however, similar, and these were largely intermediate immune or high immune groups. The inference is that any immune response is valuable in terms of prognosis. Our hypothesis is, however, that these subgroups may respond differently to immunotherapy and therefore their identification may be of value in predictive marker studies.

Tumors circumvent immune responses in a number of ways (33, 34), and it is therefore important to identify key drivers of immune activation, immune evasion, and associated biomarkers. In this paper we present tumor expression of multiple checkpoint molecules that function in health to mitigate adverse effects of excess immune response. That immune activation promotes the expression of coinhibitory signals in tumors and predicts better outcome is well described (35). Our data therefore reflect the efficacy of T cell responses in the majority, although there was some variation between tumor clusters in the patterns of expression of these molecules. In high immune CIC2 (greatest T cell scores), there was remarkable uniformity in expression of the vast majority of immune checkpoint genes, consistent with the suggestion of redundancy (36), although this has been contested (37). In intermediate immune CICs 3 and 5, accordingly, there was weaker expression of checkpoint molecules but with variation between groups: intermediate immune/keratin rich CIC5 had more *VSIR* (encoding VISTA) and *HAVCR2* (TIM-3) mRNA. In low immune/β-catenin high CIC4 (worst outcome and weak T cell infiltration), there was little evidence of immune failure as a result of primary upregulation of checkpoint molecules overall. It was notable that in the overwhelming majority of tumors in both the LMC and TCGA, multiple checkpoint molecules appeared coordinately elevated in response to T cell infiltration, adding support to recent clinical observations that combining checkpoint therapies may outperform monotherapy (38). The variation seen is, however, sufficient to argue that this variation should be tested as a potential predictive biomarker of response to checkpoint blockade.

Significant variation in expression of *ARG1* (arginase-1) and *ARG2* (arginase-2) was seen (Figure 2C). *ARG1* was mostly upregulated in keratin rich CICs 5 and 6, while *ARG2* was mostly upregulated in low immune/β-catenin CIC4. Since CICs 4 and 6 have the weakest immune responses, this observation is consistent with previous reports that arginase may perturb T cell activity by depleting the arginine in the milieu (39), thus promoting tumor growth (40, 41). However, as stated earlier, *ARG1* may be derived from the epidermis and not play a relevant immunosuppressive role. *ARG2* is not reported by the Human Protein Atlas (https://www.proteinatlas.org/) as generally expressed in melanoma cells; therefore, this signal is likely to be coming from stromal cells in low immune/β-catenin high CIC4. Arginase 2 is reported to be synthesized by cancer-associated fibroblasts (CAFs) in pancreatic cancer (42), and our data would thus suggest a possible role for CAF-derived arginase 2 in the immunosuppressive environment of low immune/β-catenin high CIC4. Because of shortage of matched tumor tissue, we were unable to explore protein expression of the arginases in this sample set.

Immune evasion via activation of β-catenin signaling was reported in human melanoma (43) using an earlier TCGA data release containing 266 metastatic melanomas. Here we have used a more recent release of TCGA data with 472 melanomas of which 103 are primaries. Furthermore, in that earlier report, samples were split into 2 groups using a T cell signature (T cell inflamed and noninflamed), while we have used the Melanoma Immunome to develop a fuller picture of the tumor microenvironment, resulting in the identification of 6 CICs with different immunophenotypes. The association between β-catenin signaling and immune signatures was replicated in this more heterogeneous TCGA data set and more notably in the LMC data set (all primaries). Importantly, mouse models have indicated that β-catenin signaling exerts immune evasion via downregulation of *CCL4* and impaired trafficking of *BATF3*-expressing CD103^+^ DCs (homologous to CD141^+^ DCs in humans), thus limiting T cell responses (26). Our study exposes this mechanism in 42% of primary melanomas overall and 73% of 1 particular subgroup with the poorest outcome (CIC4). Further mouse models have shown that DCs expressing *CCR7* play a critical role in antigen transport and T cell priming in lymph nodes (44) and that failure to recruit these DCs causes an upregulation of β-catenin signaling; our data revealed significantly reduced expression of *CCR7* in primary tumors classified as low immune/β-catenin high CIC4 (Figure 2C), further supporting the view that increased β-catenin signaling may be pivotal in controlling DC-mediated immune responses (26) and its therapeutic inhibition in conjunction with immunotherapy might be usefully explored.

The 6 CICs, their association with survival, and their characteristic immunomodulatory profiles were replicated in TCGA data where the additional methylation, copy number, and mutation data showed consistency with transcriptomic data. Collectively, these data demonstrate the existence of amplifications, putative activating mutations, hypomethylation, and ultimately higher expression of different β-catenin signaling components in the tumor cluster with the least evidence of immune response (Figure 3). Furthermore, protein data (IHC and RPPA) confirmed the observations made at mRNA level for the key genes *CTNNB1*, *CD274* (PDL-1), and *LCK*. The strength of this recapitulation is significant, as the LMC is made up of primary tumors while the TCGA melanoma data set is predominantly composed of metastases. Although we were unable to describe the cell distribution of proteins in the TCGA data set, similar results have been reported in lung cancer (45). Taken together, these data strongly suggest that genetic, epigenetic, and consequent transcriptomic modification of tumoral β-catenin signaling contributes to immune suppression in a significant proportion of skin melanomas. Our study shows that immune signatures predict outcomes at the time of diagnosis of the primary, but that they also have the same predictive value for metastatic disease. We had 9 paired samples of primary and nodal/subcutaneous metastatic tumors in this study, and metastases tended to have a weaker immune response than their paired primaries, which is not surprising. For some of them, however, data suggested that host tissue signatures were detected, of keratin in the subcutaneous metastasis and of nodal normal lymphocytes in the nodal metastases. This sample set was too small to draw strong conclusions, but the evidence suggests that nonlymphatic tissues would be preferable for metastasis immune profiling, and that metastatic tissue will likely have some signals from the host organ, which should be accounted for in their interpretation.

It has been reported in the gene, environment and melanoma (GEM; https://www.gemstudy.org/main/index.html) study that *NRAS*-mutated primaries are less likely to have brisk TILs on histopathological examination (19). Our data show that CD8^+^ T cell and NK cell scores had no protective effect in *NRAS*-mutated (codon 12, 13, or 61) tumors, some effect in *BRAF*-V600–mutated tumors, and the greatest effect in double-WT tumors, meaning that even when T cells are present they are much less effective in terms of survival in the presence of *NRAS* mutations. Since *NRAS* mutations are more frequent in tumors from older males, we initially suspected that these factors confounded the lack of association, but it persisted when we adjusted for age and sex, and we made similar observations in TCGA data. Some reports have suggested that *NRAS* mutations might predict a better response to immunotherapy and that *NRAS*-mutated tumors may express more PDL-1 (46). More recently, it was reported (47) that patients with *BRAF*-mutated melanoma were less likely to require combined PD-1 and CTLA4 blockade than others, reinforcing the view that mutation status may predict response to checkpoint blockade. Our data do not support PDL-1 expression as a mechanism explaining a differential response to T cell infiltration in tumors with different driver mutations.

In summary, we report the immune landscape of a large population-ascertained sample set of primary melanomas with replication in primary and metastatic disease. We identified subgroups with gene expression patterns indicative of different immune responses confirmed by pathologist-reported TILs. In 42% of primary melanomas overall and 73% of those with the poorest outcome, we report a strong confirmation of a causal role for β-catenin signaling in immune response failure, resulting from epigenetic modifications, activating mutations, and DNA amplification. These data suggest that β-catenin signaling should be an important therapeutic target for the future and that immunotherapy clinical trials should be stratified on driver mutations.

## Methods

### Transcriptomic data generation and preprocessing.

FFPE melanoma tumor blocks were obtained from the population-ascertained Leeds Melanoma Cohort (LMC) (10, 11). Two thousand eighty-four patients were ascertained from pathology and clinical registers in a geographically defined area of the northern part of the United Kingdom, with additional recruitment from 32 other clinical centers carrying out sentinel node biopsies (total 342 recruits) and rare subtypes of cases with melanomas arising in sun-protected sites (total 76 recruits). Patients were invited to participate at 3 months after diagnosis with the intent of interviewing and sampling them within the period 3–6 months after diagnosis. Patients responded variably quickly, and the median time to interview was 5.2 months. Seven hundred three of 2,184 tumors were sampled (see below). The median follow-up time at the end of this analysis is 7.5 years. Only 10 participants whose transcriptomes were analyzed have to date received BRAF inhibitors, 10 ipilimumab and 2 pembrolizumab, so the data reflect essentially treatment-naive patients. Histopathological factors associated with survival such as Breslow thickness and ulceration status were as clinically reported. Three measures of tumor-infiltrating lymphocytes (TILs) were used: first, TILs as classified by Clark et al. (48) and recently clarified by Schatton et al. (1) and reported by specialist histopathologists in the clinical service; second, TILs scored by a blinded single observer in the research group (S. O’Shea)in the whole tumor slide; and third, TILs also graded by a single observer (S. O’Shea) in the area of the tissue cored for RNA extraction. These were scored with a 4-grade score from “lots” to “none/barely perceptible.” Follow-up data were ascertained by examination of hospital medical notes, primary care records, annual questionnaires from consenting participants, and cancer registries, and melanoma-specific survival data were derived.

The tumor blocks were sampled horizontally using a 0.6-mm microarray needle as marked by J. Newton-Bishop. J. Newton-Bishop reviewed all H&E sections of primary tumors and marked deep invasive tumor, which was the least stromally rich and least inflamed, to allow comparison within the data set. Where large tumors were available the ethical approval allowed sampling using up to 2 cores, which were chosen to be most similar areas of the tumor. The mRNA was extracted from 820 tumor cores (703 unique patients and 117 duplicates) as previously described (49, 50), and gene expression was quantified using the Illumina DASL Human HT12 v4 array. The data were deposited into the European Genome-phenome Archive (EGA) (accession no. EGAS00001002922). Paired sample transcriptomes were available from 9 patients: 9 paired primary and soft tissue metastases.

GenomeStudio was used to extract raw data from the image files. Data were background-corrected and quantile-normalized using the package Lumi (51) in R. Singular value decomposition (SVD) was applied in Swamp R package (52) to assess the association between top principal components and technical variables such as batch, chip, age of FFPE block, and RNA concentration. Technical variables found to be associated with these top components were adjusted out, and SVD was reapplied with and without data permutation to appraise the remaining “biological” variability in the data. Normalized full-intensity plots were examined to detect outliers. Among sample duplicates, we retained in the final data set the sample with the highest number of detected genes. Overall, the median and interquartile range of detected genes per sample at *P* less than 0.05 were 14,784 (range 14,153–15,304), consistent with other studies using DASL arrays in melanoma (14, 53–55). After quality control, genes were standardized to a mean 0 and variance 1, and samples were randomly split into a training set (two-thirds) and a test set (one-third). Subsequently, when the analysis revealed similar results between the training and test sets, the total pooled data set was used in downstream analyses.

### Derivation of Melanoma Immunome.

Bindea et al. (8) derived the Immunome Compendium from their own and published data on genes expressed by FACS-sorted immune cells. Genes that appeared to be expressed only by specific immune cell subsets were used to define the presence of those cell subtypes in the tumor. A total of 577 genes was included in the Immunome Compendium, encompassing those specific to each of 24 immune cell types and some control tissues: SW480 colon cancer cell line, normal mucosae, blood, and lymphatic vessels (8). From this list, we excluded genes expressed by these control tissues, and we further eliminated all genes that we found highly expressed in melanoma cell lines SK-MEL-28 (ATCC HTB-72) and MEWO (ATCC HTB-65) cultured in house and in published melanocyte cultures (Gene Expression Omnibus [GEO] GSE4570). In each of these cell lines we considered as highly expressed any gene whose expression level ranked in the top 25% relative to the whole genome. The remaining genes (*n* = 380) and the cell types they represent (*n* = 24) after this filtering are referred to in this paper as the Melanoma Immunome. The following abbreviated names were used to represent the cell types: iDC/aDC/pDC, immature/activated/plasmacytoid DC; Tem, effector memory T cell; Tcm, central memory T cell; Tfh, follicular helper T cell; Tgd, γδ T cell; NKd, natural killer CD56^dim^ cell; and NKb, natural killer CD56^bright^ cell. An important feature of the original Immunome Compendium (8), and therefore also of the Melanoma Immunome, is that certain genes are specific not to a particular cell type but to several subtypes or a broader type of cell. For example, genes expressed by both NKb and NKd defined the broader NK cell phenotype. Genes expressed by all NK cell subtypes, CD8^+^ T cells, and Tgd cells were labeled cytotoxic genes, and genes expressed by various T cell subtypes (Th1, Th2, CD8^+^ T cell, Tem, Tcm) were labeled as broader T cell genes. This system allows scoring of generic cell types in addition to their more specific subtypes.

### Tumor classification using the Melanoma Immunome.

Using the Melanoma Immunome, we classified tumor samples using the consensus cluster approach (12) in the training data set with the R package ConsensusClusterPlus (13). We used the KMeans algorithm with a maximum number of 12 clusters, the Euclidian distance, 80% genes and tumor resampling, and 5,000 repeats. The final number of clusters was determined by examination of consensus cluster matrices, plots of consensus cumulative density function (CDF), and the change in the area under the CDF curve, i.e., ΔCDF (13). It is important to note that this clustering approach aimed to identify the best tumor groupings and did not generate gene clusters. Simply, then, to allow graphical representation, after choosing the optimal number of tumor clusters (consensus immunome clusters, or CICs), we also clustered the genes (1-pass without resampling) while fixing samples in their respective CICs. For this gene clustering analysis, we again used the KMeans algorithm and fixed K = 4, i.e., 4 gene clusters. This number was chosen to reflect 2 major immune responses, innate and adaptive, and their putative components with good or bad effect on outcome (56). The obtained tumor clusters in the training data set were replicated in the test set using the supervised nearest centroid method (53, 57) with the Spearman correlation similarity metric.

### Scoring each cell subtype, innate, adaptive, and total immune function.

We assigned a simple score to each cell type in each tumor as an average of the expression of all genes specific to that cell type, after standardizing the expressions to mean 0 and variance 1. We then rescaled each cell score to a range of 0–1. To obtain the total immune score per tumor, we added together scores from all 24 cell subtypes. For innate and adaptive scores, we added immune scores from each type according to Bindea et al. (8): NK, NKb, NKd, DC, aDC, iDC, pDC, mast cells, macrophages, eosinophils, and neutrophils for innate immunity; B cells, T cells, CD8^+^ T cells, cytotoxic cells, Th, Tfh, Th1, Th2, Th17, Treg, Tcm, Tem, and Tgd for adaptive immunity. To evaluate this scoring scheme, we plotted key immune cell scores alongside the CICs, and the innate versus the adaptive immune score, and compared the total score with an equivalent score calculated using the published software ESTIMATE (18). Similarly, we plotted this total immune score alongside the molecular phenotypes inferred in our data using 2 published gene signatures by Jonsson et al. (14) and TCGA (15).

### Statistical analyses of the CICs and immune scores.

Kaplan-Meier plots and Cox proportional hazards regression were used to analyze the difference in survival between the CICs. We compared the CICs for clinical melanoma characteristics using the Kruskal-Wallis test for continuous variables and χ^2^ or Fisher’s exact tests for categorical variables. Multivariable survival analysis was conducted, testing the joint effects of the CICs and up to 8 histological variables (age, sex, tumor site, AJCC stage, Breslow thickness, ulceration, mitotic rate, vascular invasion, *BRAF* and *NRAS* mutation status) in the Cox model. We also tested the added prognostic value of the CICs compared with the expression of *CD8A* and *CD8B* in Cox proportional hazards model, i.e., whether the CICs are a simple reflection of a T cell function. The association between immune cell scores and genes coding for a range of checkpoint/inhibitory molecules was assessed and represented graphically as a heatmap. We considered genes coding for the most studied immune checkpoint coinhibitors: programmed cell death 1 (*PD-1*), its ligands *PDL-2* and *PDL-1* (also known as *CD274*), and cytotoxic T lymphocyte–associated protein 4 (*CTLA4*); along with a representative set of other negative regulators of immune responses emerging as new targets of immunotherapies currently in clinical trials: T cell immunoglobulin and mucin-domain containing-3 (*TIM-3*; also called hepatitis A virus cellular receptor 2 [*HAVCR2*]), lymphocyte activation gene 3 (*LAG3*), and V-domain immunoglobulin-containing suppressor of T cell activation (VISTA; encoded by the *C10orf54* gene) as well as B and T lymphocyte attenuator (*BTLA*) (58–61). We also included the immunosuppressive enzymes indoleamine 2,3-dioxygenase 1 and 2 (*IDO1*, *IDO2*), which are being tested as immunotherapy targets in clinical trials (62), and expanded our list to other immunological targets such as *CD40*, *CD200*, *TNFRSF4*, *TNFRSF18*, *TNFRSF9*, *CD72*, *CD27*, *ARG1*, *ARG2*, *KIR3DL1*, and *ICOS*.

To investigate immune cell association with the β-catenin signaling pathway, we plotted the expression of key genes from this pathway (43) on the cell scores heatmap. There is published evidence that when expressing *CCR7*, CD141^+^ DCs drive intratumoral T cell activation (44), and that failure to recruit these cells is causally related to increased β-catenin signaling. To assess whether these mechanisms would be detectable in archived primary tumors, we assessed the association between expression of *IRF8*, *BATF3*, and *CCR7*, DC score, T cell score, and expression of genes from the β-catenin signaling pathway. To derive the proportion of tumors with evidence of upregulated β-catenin expression in this population cohort, we used the means test of X-tile (17) to generate an optimal cutoff value of *CTNNB1* that better predicts the variation in T cell score. We also generated a score for the β-catenin signaling pathway as sum of standardized expressions of *CTNNB1*, *c-MYC*, *APC*, *APC2*, *SOX2*, *SOX11*, *TCF1*, *TCF12*, and *VEGFA* (43), i.e., *gx*_score_ = ∑*gx*, where *gx* = standardized gene expression and the sum is over the 9 genes listed.

### Replication in TCGA.

We downloaded The Cancer Genome Atlas cutaneous melanoma data from cBioPortal (http://www.cbioportal.org/data_sets.jsp), including RNA-Seq expression, copy number alterations, DNA promoter methylation, and the mutations. To replicate the cluster analysis, each tumor was assigned to one CIC by application of the nearest centroid method (53, 57) to RNA-Seq data. Cox proportional hazards regression was used in overall survival analysis comparing the CICs, unadjusted and adjusting for other prognostic factors. Association of the CICs with expression of genes encoding checkpoint coinhibitors, immunomodulatory enzymes, and transcription factors and β-catenin signaling was conducted in the same way as in the LMC. As a sensitivity analysis, we combined TCGA primary tumors (*n* = 103) with the total LMC sample (all primaries) and conducted a new consensus cluster analysis with the same parameters (80% gene and tumor resampling, KMeans algorithm with K = 12 and Euclidian distance, 4 gene clusters) and compared the results with the initial analysis. Gene promoter methylation data were compared with RNA-Seq data for the β-catenin pathway genes to assess the epigenetic contribution to gene expression. Copy number alterations and point mutations were plotted alongside gene expression and the CICs for genes in the β-catenin signaling pathway using the OncoPrint graph function (63) to assess the role of structural modification in the regulation of immune evasion. The genes for OncoPrint representation were chosen based on frequency of mutations and alterations with a cutoff of 4%. Enrichment in these genetic alterations and mutations within a particular CIC compared with the others was tested using Fisher’s exact test. β-Catenin pathway score was generated as in the LMC data set using the expression of 9 genes (*gx*_score_). The pathway methylation score was generated as methyl_score_ = ∑(1 – β), where β = proportion of methylated CpG sites. The mutation and copy number score was generated as mutCNV_score_ = ∑(*I* + *J*), where *I* = 1 if the gene has a putative activating mutation and 0 otherwise, and *J* = –1 if the gene is deleted, +1 if it is amplified, and 0 otherwise. In each of these scores, the sum is over the 9 genes (*CTNNB1*, *c-MYC*, *APC*, *APC2*, *SOX2*, *SOX11*, *TCF1*, *TCF12*, and *VEGFA*). To test additive value of these different data types, these scores were combined (*gx*_score_ + methyl_score_, then *gx*_score_ + methyl_score_ + mutCNV_score_), and their variation across tumor clusters was evaluated.

### IHC staining and protein data validation.

In a subset of the LMC, we ran the IHC staining for β-catenin (33 tumors) and its membranous ligand E-cadherin (37 tumors). Under the terms of our study ethical approval and the consent given by the participants, we had access only to stored tumor blocks from participants who had died of melanoma or other causes (the intent being to conserve tumor blocks for clinical testing). The tumor set examined was therefore small and somewhat biased in favor of poor-prognosis tumors. The slides were therefore used just to compare gene expression results with level of protein expressed.

### Staining.

After the melanoma FFPE blocks were sampled, 5-μm sections were cut and mounted onto Superfrost Plus slides (Thermo Fisher Scientific) and underwent IHC staining using IntelliPath FLX detection reagents (MenaPath, A. Menarini Diagnostics). Antigen retrieval by heat was performed in Access Revelation (pH 6.4), followed by peroxidase quenching and background blocking with casein. Tissue sections were incubated with antibodies raised against human β-catenin (9562, New England Biolabs) or E-cadherin (14472, New England Biolabs) followed by MenaPath HRP-polymer or Universal Probe and HRP-polymer, respectively. IHC labeling was detected using MenaPath purple chromogen.

### Scoring.

Light microscopy (×10 magnification) was used to assess expression of β-catenin and E-cadherin. Staining scores for the regions immediately surrounding the tumor core “punch hole” were recorded. In those tumors that were cored twice (and hence had 2 punch holes), 2 staining scores were generated; where these were discordant, the slides were not used for subsequent analyses. For β-catenin, 3 staining scores were measured as determined by the cellular localization — overall, cytoplasmic, and membranous. Each was assessed on a scale of 0–3 and was used to measure both the intensity and the distribution of staining surrounding the cored tumor region (0, no staining; 1, weak staining; 2, intermediate staining; 3, high intensity and distribution of staining). For E-cadherin, the membranous staining intensity was homogenous for most tumors and was attributed a score based on the distribution of staining on a scale of 0–3 (0, no staining; 1, weak staining; 2, intermediate staining; 3, high distribution). Statistical analyses were conducted comparing the level of staining and mRNA expression (Kruskal-Wallis test). When 2 or more consecutive levels of staining showed no difference between them, they were pooled together to have fewer categories and reduce random variation. Scoring was developed by pairs of observers (J. Newton-Bishop and 1 other) and standards identified. J. Newton-Bishop and 1 other then scored slides separately (blind to gene expression status), and differences were resolved.

We also downloaded protein-level data (Reverse Phase Protein Array, RPPA; http://tcpaportal.org/tcpa/download.html) for 354 of the 472 TCGA skin melanomas. RPPA measures protein levels on a continuous scale, and we selected the key immune genes *LCK* and *CD274* (PDL-1) as well as β-catenin and E-cadherin to assess the correlation between protein-level data and mRNA expressions. We also tested the difference in protein-level data between tumor clusters.

### Statistics.

A range of analyses are described in the relevant sections of Methods. Where required, confounding factors were adjusted for, and multiple testing was dealt with by Bonferroni correction. Nonparametric methods (Kruskal-Wallis, Mann-Whitney, Fisher’s exact test, and Spearman correlation) were used; 1-way ANOVA was used to estimate the proportion of variance explained. Cox regression and log-rank tests were used in survival analyses. Two-sided tests were applied throughout, and a *P* value below 0.05 (after multiple-testing adjustment where required) was considered significant.

### Study approval.

The Leeds Melanoma Cohort was reviewed by the North East – York Research ethics committee (Jarrow, Tyne and Wear, UK) and received ethical approval MREC 1/3/57, PIAG 3-09(d)/2003. All participants gave written informed consent prior to their participation in the study.

## Author contributions

Design was contributed by DTB, JNB, and JN. Recruitment was contributed by JNB, CW, JG, and LP. Data management was contributed by MC and JG. Tumor sampling was contributed by JL, AF, RJ, JRM, MP, and JNB. IHC staining was contributed by JL, TM, JP, SM, and JNB. TIL evaluation was contributed by SO. Analysis and interpretation was contributed by JN, AD, JP, SM, JNB, GPC, and DTB. All authors contributed to the writing of the manuscript.

## Supplementary Material

Supplemental data

## Figures and Tables

**Table 6:**
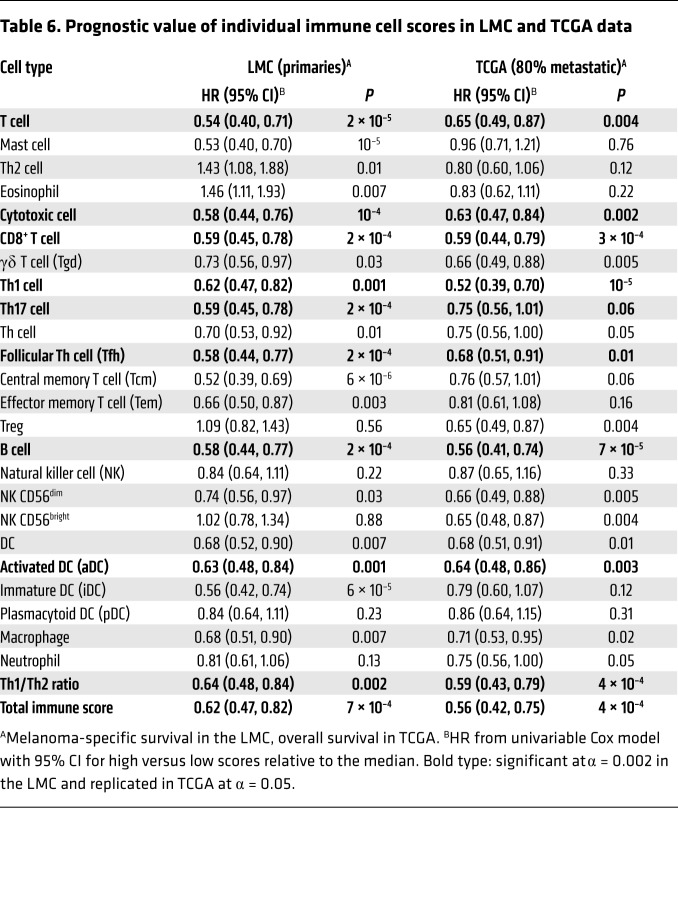
Prognostic value of individual immune cell scores in LMC and TCGA data

**Table 5:**
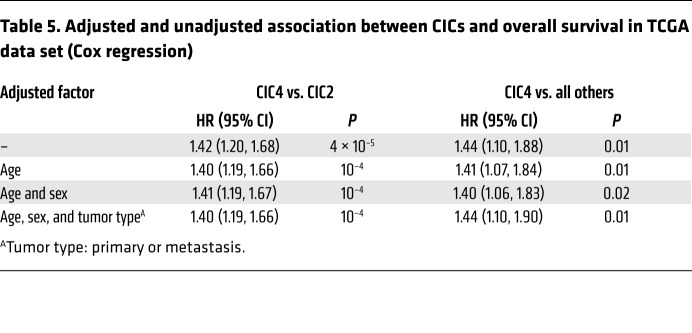
Adjusted and unadjusted association between CICs and overall survival in TCGA data set (Cox regression)

**Table 4:**
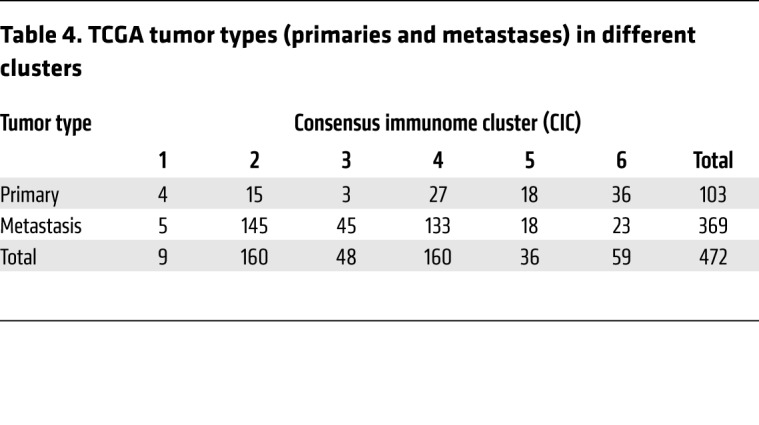
TCGA tumor types (primaries and metastases) in different clusters

**Table 3:**
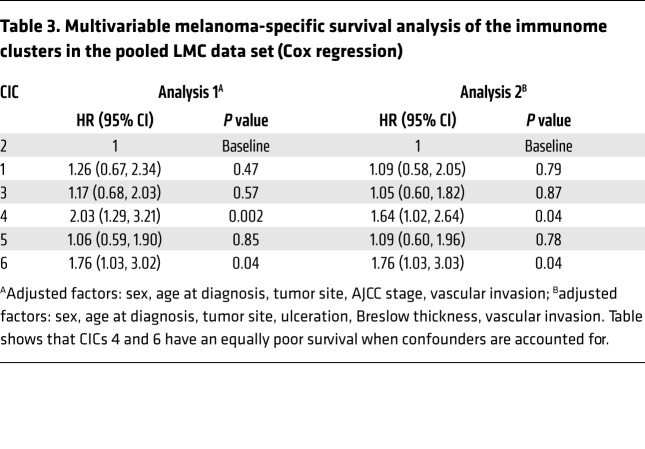
Multivariable melanoma-specific survival analysis of the immunome clusters in the pooled LMC data set (Cox regression)

**Table 2:**
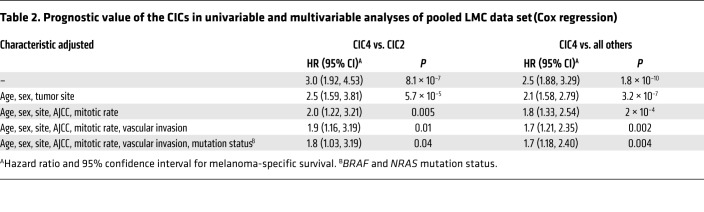
Prognostic value of the CICs in univariable and multivariable analyses of pooled LMC data set (Cox regression)

**Table 1:**
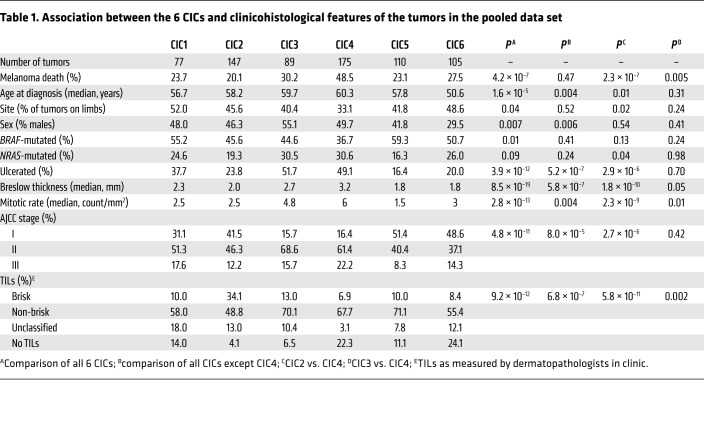
Association between the 6 CICs and clinicohistological features of the tumors in the pooled data set

## Version 1 04/16/2018

Electronic publication

## Version 2 05/01/2018

Print issue publication
