# Supplementary material for: β-Catenin–mediated immune evasion pathway frequently operates in primary cutaneous melanomas
Source: J Clin Invest. 2018 Apr 16;128(5):2048–63. doi: 10.1172/JCI95351 (PMC5919828; doi:10.1172/JCI95351)
Supplement: Supplemental data [file jci-128-95351-s001.pdf]

**Supplementary data:**

# β-catenin-mediated immune evasion pathway frequently operates in primary cutaneous melanomas

Jérémie Nsengimana,[1] Jon Laye,[1] Anastasia Filia,[1,2] Sally O'Shea,[1] Sathya Muralidhar,[1] Joanna Poźniak,[1] Alastair Droop,[1,3] May Chan,[1] Christy Walker,[1] Louise Parkinson,[1] Joanne Gascoyne,[1] Tracey Mell,[1] Minttu Polso,[1] Rosalyn Jewell,[1,4] Juliette Randerson-Moor,[1] Graham P Cook,[1] D Timothy Bishop,[1] and Julia Newton-Bishop[1]

1. Leeds Institute of Cancer and Pathology, University of Leeds School of Medicine, Leeds, UK
2. National Heart and Lung Institute, Imperial College, London, UK
3. MRC Medical Bioinformatics Centre, University of Leeds, UK
4. Yorkshire Regional Genetics Service, Leeds Teaching Hospitals NHS Trust, Leeds, UK

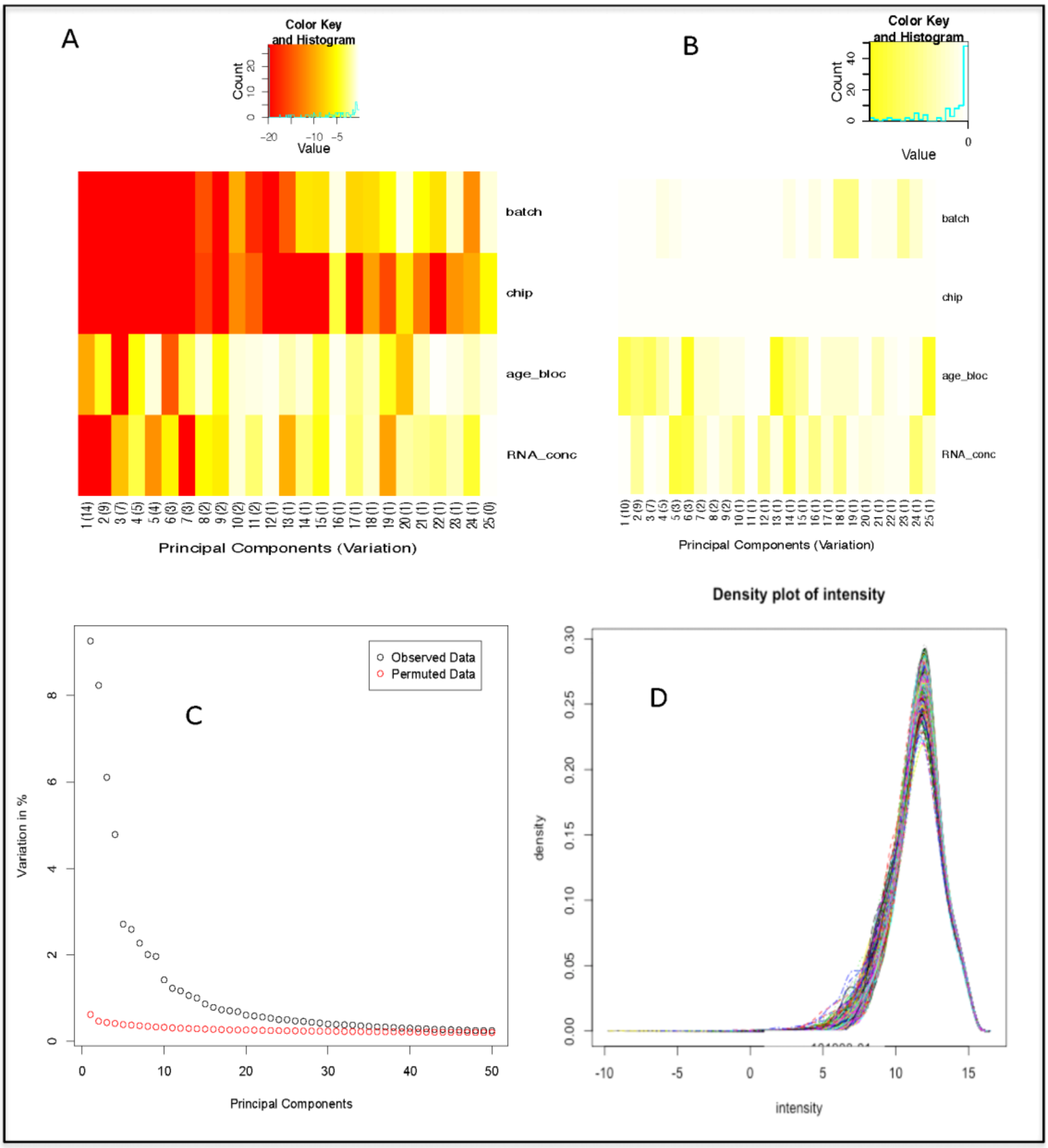


**Figure S1**. Leeds Melanoma Cohort (LMC) whole genome transcriptomic data quality control. **A**. Heatmap showing a strong correlation between the top 25 principal components of the whole transcriptome and technical variables batch, chip, age of the FPPE block and RNA concentration. Red color indicates highly significant correlation [log10(P value) down to -20]. **B**. After adjusting out the effect of the chip, no principal component correlated with technical variables. **C**. The proportion of variance explained by the top 50 principal components after chip adjustment remained much higher than that of permuted data (average of 1000

permutations), indicating that biological variation remained intact. **D**. Normalised density plots.

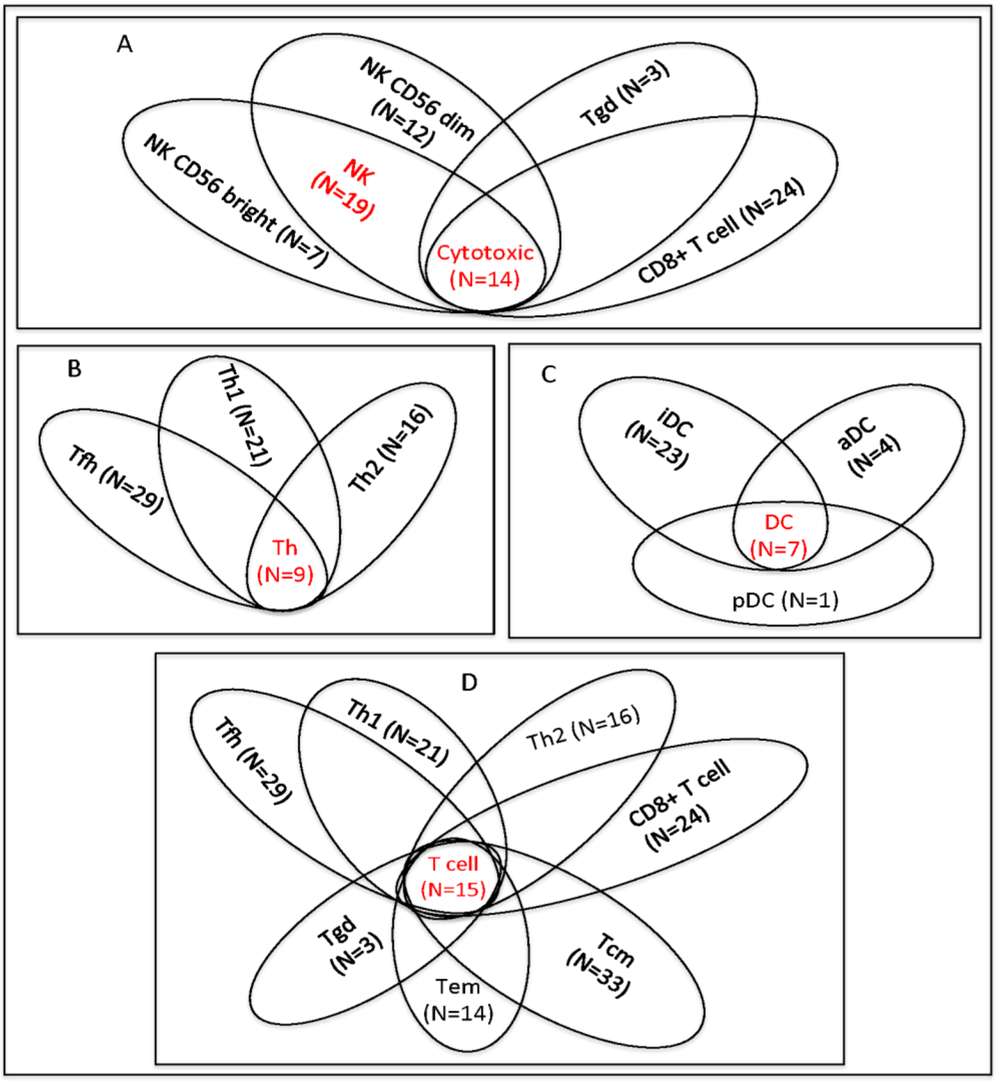


**Figure S2**. Schematic representation of overlapping immune cell subtypes defined in Bindea *et al* (1). Shown in brackets are the numbers of genes of each cell subtype after applying our filters (see also Table S2). **A**. Genes expressed by CD56$^{dim}$ and CD56$^{bright}$ Natural Killer cells were defined the broader NK (e.g. *BCL2, MRC2, …).* Genes expressed by NK, gamma-delta T and CD8 + T-cells defined the cytotoxic cells (e.g. *KLRB1, KLRD1, GZMH, GZMA, ….*) **B**. Genes expressed by 3 types of helper T-cells defined the broader helper T-cell (Th). They included e.g. *ICOS, CD28, BATF,* … **C**. Genes expressed by activated, immature and plasmacytoid DCs defined the broader DC subset (e.g. *CCL17*, *CCL22*, *CCL13*, *CD209*, …). **D**. Genes expressed by various types of T and helper T-cells defined the broader T-cell (e.g. *LCK, CD3G, CD3D, CD3E, TRAT1, …).*

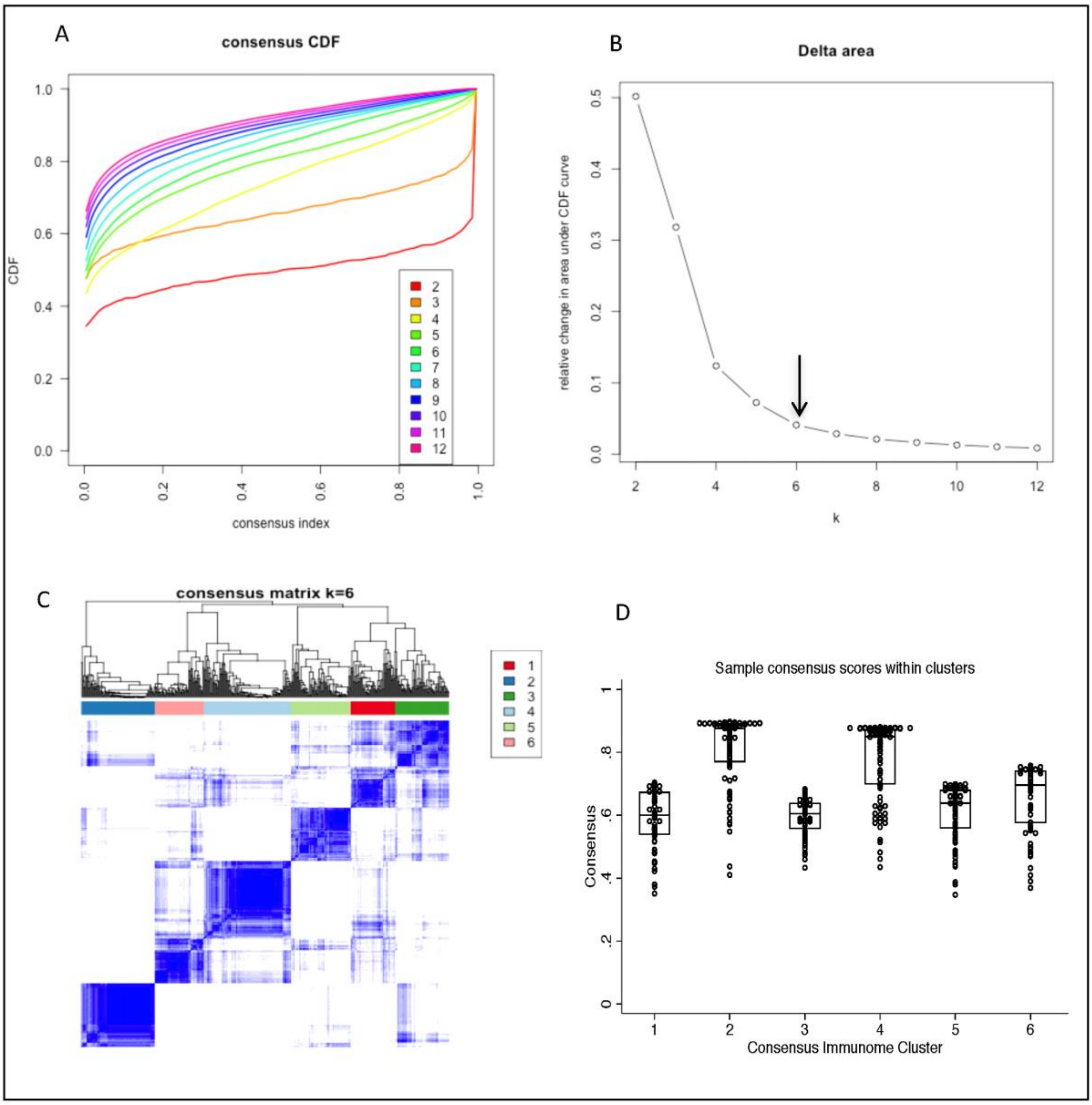


**Figure S3**. Consensus cluster analysis (2) conducted in 2/3 of LMC dataset. **A**. Cumulative density functions (CDF) of independent runs considering 2 to 12 clusters in 5000 data resampling. **B**. Relative change in area under the CDF with increasing number of tumor clusters (k). This area shows little variation after k=6 and we chose this number as optimal (shown by an arrow). **C**. Sample dendrogram with k=6. The diagram is symmetrical and blue color indicates high consensus (i.e. samples occurring in the same cluster with high frequency in the 5000 repeats) while white indicates no consensus (samples never classified together). **D**. Sample consensus scores within the 6 optimal clusters. Cluster 2 and 4 have the greatest consensus but all clusters have a median consensus > 0.63.

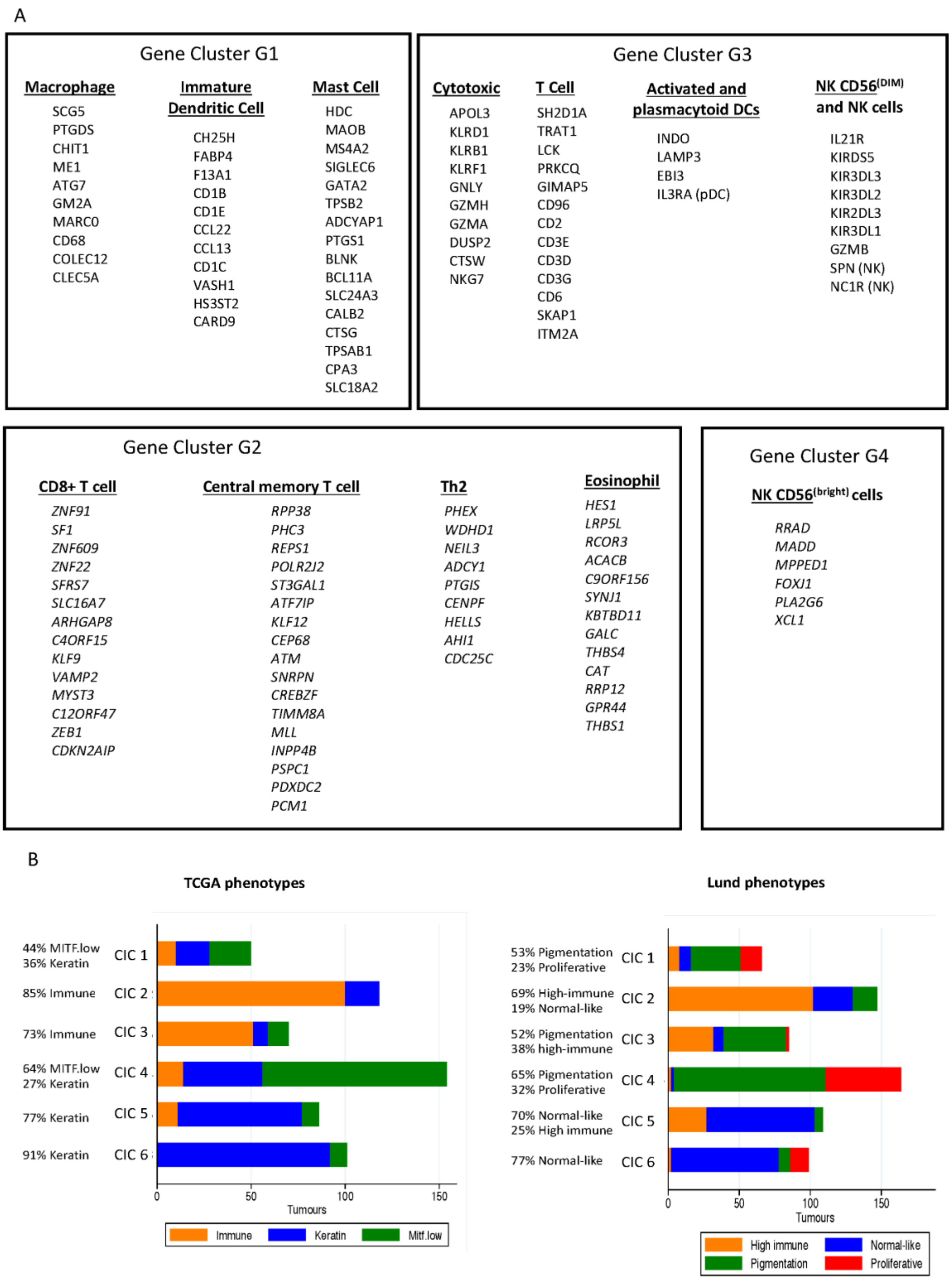


**Figure S4**. A. Overrepresentation in gene clusters G1-G4 of Figure 1A of the main txt. B. Overlap between the 6 CICs in the LMC dataset (training and test sets pooled) and molecular phenotypes inferred using two published transcriptomic signatures developed using predominantly metastatic tumors (see references in the main text).

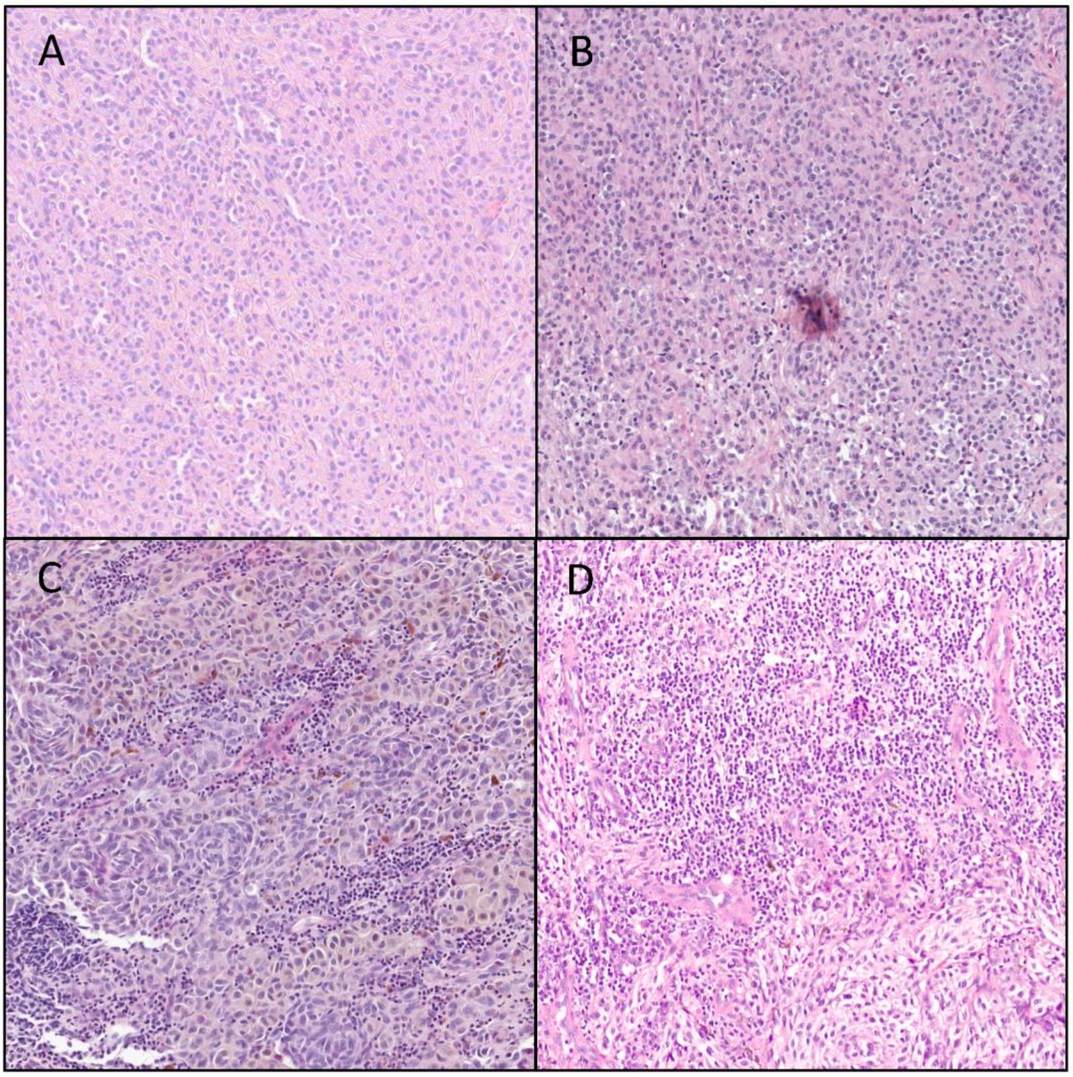


**Figure S5**. Representative images of H&E-stained primary melanomas from sectioned FFPE blocks in the LMC. Blocks were examined and annotated to identify an appropriate region for mRNA sampling for transcriptomic profiling. Retrospectively the H&Es were scored for the presence of tumour infiltrating lymphocytes (TILs) in the exact location corresponding to the sampled region of the block. Tumors were scored as containing (A) ‘no’ TILs; (B) ‘some’ TILs; (C) ‘moderate’ TILs and (D) ‘lots’ of TILs.

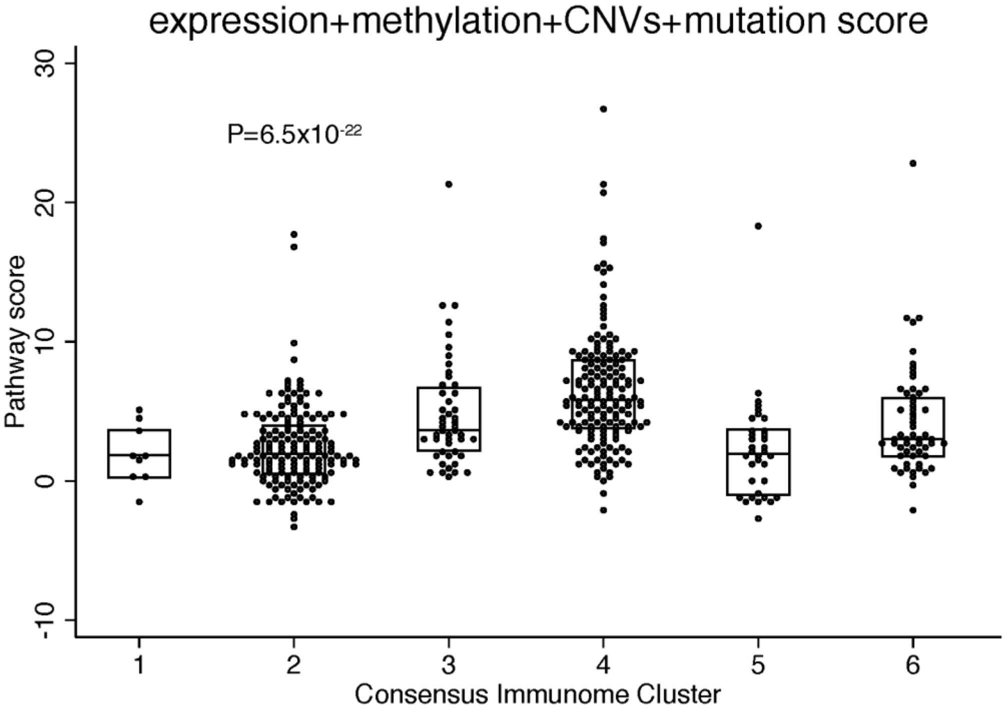


**Figure S6**. Distribution of β-catenin signaling pathway score across the 6 Consensus Immunome Clusters in TCGA skin melanoma dataset. The pathway scoring used gene expression, promoter methylation, driver mutations and copy number alterations for *CTNNB1, c-MYC, APC, APC2, SOX2, SOX11, TCF1, TCF12* and *VEGFA* (see Methods). The Pvalue is from a Kruskall-Wallis test.

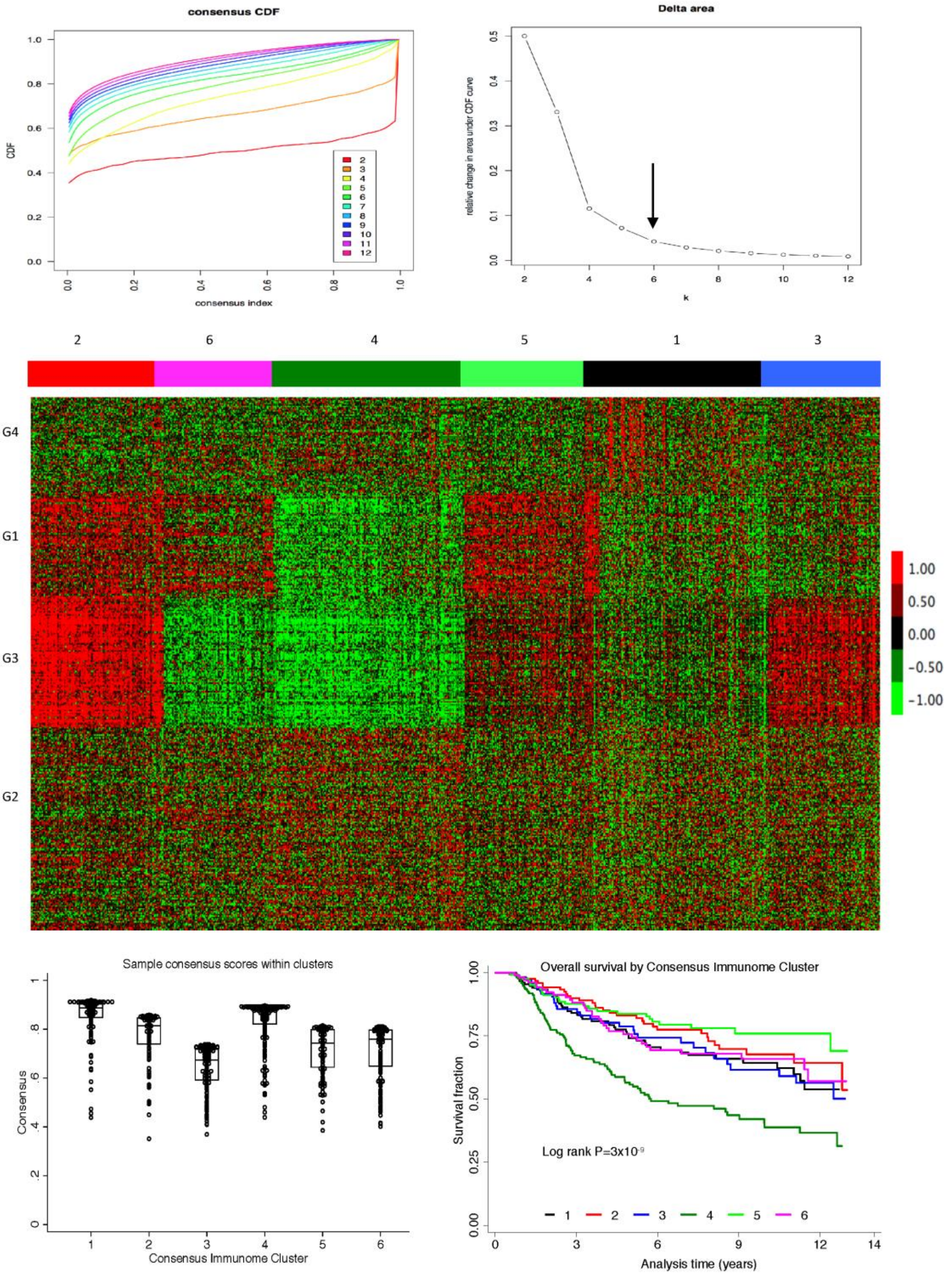


**Figure S7.** New consensus cluster analysis pooling the full LMC dataset and TCGA primaries. The consensus CDF, optimal number of clusters, heatmap and cluster association with survival are similar to those obtained in the original analysis based on 2/3 of LMC as training set.

**Table S1. Sample histological characteristics in the training and test LMC data subsets**

| | Number and percentage or median and range * | | |
|---|---|---|---|
| **Characteristic** | **Training (N=465)** | **Test (N=238)** | **P** |
| Male sex | 216 (46.5%) | 102 (42.9%) | 0.37 |
| Age at diagnosis (years) | 58.4 (18.3, 81.2) | 58.1 (20.3, 77.1) | 0.54 |
| Tumor site | | | |
| Limbs | 195 (42.0%) | 103 (43.3%) | 0.64 |
| Trunk | 161 (34.7%) | 72 (30.2%) | |
| Head/neck | 51 (11.0%) | 29 (12.2%) | |
| Other | 57 (12.3%) | 34 (14.3%) | |
| Died from melanoma | 157 (33.8 %) | 76 (31.9%) | 0.63 |
| Relapsed | 167 (35.9%) | 76 (31.9%) | 0.29 |
| Follow up time (years) | 7.6 (0.8, 13.4) | 7.5 (0.5, 13.6) | 0.73 |
| AJCC stage | | | |
| I | 158 (34.3%) | 75 (32.0%) | 0.41 |
| II | 238 (51.6%) | 117 (50.0%) | |
| III | 65 (14.1%) | 42 (18.0%) | |
| Ulcerated | 154 (33.1%) | 81 (34.0%) | 0.81 |
| Mitotic rate (count per $mm^2$) | 3 (0, 83) | 3 (0, 70) | 0.78 |
| Breslow thickness (mm) | 2.3 (0.3, 20) | 2.5 (0.7, 15) | 0.87 |
| Vascular invasion | 51 (12.4%) | 18 (8.4%) | 0.13 |
| *BRAF-V600* mutation | 183 (48.4%) | 90 (44.1%) | 0.32 |
| *NRAS* mutation (codon 12,13 or 61) | 93 (24.9%) | 49 (24.5%) | 0.92 |
| TILs | | | |
| Brisk | 55 (14.3%) | 27 (16.0%) | 0.42 |
| Non-brisk | 240 (62.5%) | 101 (60.0%) | |
| Unclassified | 41 (10.7%) | 13 (7.7%) | |
| Absent | 48 (12.5%) | 28 (16.6%) | |

* Missing data excluded in percentage calculation

[&] Rare site=non-exposed to the sun such as anal, perineal, penile, vulvar or oropharyngeal

**Table S2: Melanoma immunome gene and cell list**

| GENE | PROBE | IMMNUNE CELL | FUNCTION |
|---|---|---|---|
| ABCB4 | ILMN_1767349 | B-cells | adaptive |
| FCRL2 | ILMN_1791329 | B-cells | adaptive |
| SCN3A | ILMN_1657591 | B-cells | adaptive |
| GLDC | ILMN_1806754 | B-cells | adaptive |
| CD72 | ILMN_1723004 | B-cells | adaptive |
| QRSL1 | ILMN_1733364 | B-cells | adaptive |
| CR2 | ILMN_2369666 | B-cells | adaptive |
| SPIB | ILMN_2143314 | B-cells | adaptive |
| COCH | ILMN_1711514 | B-cells | adaptive |
| CD19 | ILMN_1782704 | B-cells | adaptive |
| MS4A1 | ILMN_1697830 | B-cells | adaptive |
| BACH2 | ILMN_1659943 | B-cells | adaptive |
| SLC15A2 | ILMN_2211739 | B-cells | adaptive |
| BLK | ILMN_1668277 | B-cells | adaptive |
| OSBPL10 | ILMN_1669497 | B-cells | adaptive |
| BLNK | ILMN_2142935 | B-cells | adaptive |
| TNFRSF17 | ILMN_1768016 | B-cells | adaptive |
| HLA-DQA1 | ILMN_1808405 | B-cells | adaptive |
| HLA-DOB | ILMN_1700428 | B-cells | adaptive |
| GNG7 | ILMN_1728107 | B-cells | adaptive |
| PNOC | ILMN_1676003 | B-cells | adaptive |
| DTNB | ILMN_1798819 | B-cells | adaptive |
| CCR9 | ILMN_1664316 | B-cells | adaptive |
| TCL1A | ILMN_1788841 | B-cells | adaptive |
| BCL11A | ILMN_1752899 | B-cells | adaptive |
| GZMM | ILMN_1690561 | CD8 T-cells | adaptive |
| VAMP2 | ILMN_1713491 | CD8 T-cells | adaptive |
| ZFP36L2 | ILMN_2150258 | CD8 T-cells | adaptive |
| MYST3 | ILMN_2095840 | CD8 T-cells | adaptive |
| ZNF22 | ILMN_1798533 | CD8 T-cells | adaptive |
| KLF9 | ILMN_1778523 | CD8 T-cells | adaptive |
| PRF1 | ILMN_1740633 | CD8 T-cells | adaptive |
| ZEB1 | ILMN_1762231 | CD8 T-cells | adaptive |
| ARHGAP8 | ILMN_2297096 | CD8 T-cells | adaptive |
| SFRS7 | ILMN_1778836 | CD8 T-cells | adaptive |
| ZNF91 | ILMN_1802053 | CD8 T-cells | adaptive |
| CD8A | ILMN_1760374 | CD8 T-cells | adaptive |
| LIME1 | ILMN_2183687 | CD8 T-cells | adaptive |
| SF1 | ILMN_1742808 | CD8 T-cells | adaptive |
| CD8B | ILMN_1748601 | CD8 T-cells | adaptive |
| SLC16A7 | ILMN_1810053 | CD8 T-cells | adaptive |
| FLT3LG | ILMN_2091412 | CD8 T-cells | adaptive |
| ZNF609 | ILMN_1799082 | CD8 T-cells | adaptive |
| PF4 | ILMN_1767037 | CD8 T-cells | adaptive |

| | | | |
|---|---|---|---|
| TSC22D3 | ILMN_2276952 | CD8 T-cells | adaptive |
| PPP1R2 | ILMN_1683044 | CD8 T-cells | adaptive |
| C12orf47 | ILMN_1798957 | CD8 T-cells | adaptive |
| CDKN2AIP | ILMN_1773066 | CD8 T-cells | adaptive |
| C4orf15 | ILMN_2160124 | CD8 T-cells | adaptive |
| GZMA | ILMN_1779324 | Cytotoxic cells | adaptive |
| KLRD1 | ILMN_1797988 | Cytotoxic cells | adaptive |
| KLRB1 | ILMN_2079655 | Cytotoxic cells | adaptive |
| GNLY | ILMN_1790692 | Cytotoxic cells | adaptive |
| CTSW | ILMN_1794364 | Cytotoxic cells | adaptive |
| APBA2 | ILMN_1723626 | Cytotoxic cells | adaptive |
| KLRK1 | ILMN_2222443 | Cytotoxic cells | adaptive |
| DUSP2 | ILMN_1712959 | Cytotoxic cells | adaptive |
| APOL3 | ILMN_1756862 | Cytotoxic cells | adaptive |
| RORA | ILMN_2322499 | Cytotoxic cells | adaptive |
| ZBTB16 | ILMN_2305407 | Cytotoxic cells | adaptive |
| GZMH | ILMN_1731233 | Cytotoxic cells | adaptive |
| KLRF1 | ILMN_2055781 | Cytotoxic cells | adaptive |
| NKG7 | ILMN_1682993 | Cytotoxic cells | adaptive |
| FRYL | ILMN_1798467 | T helper cells | adaptive |
| ATF2 | ILMN_1748271 | T helper cells | adaptive |
| ICOS | ILMN_1669927 | T helper cells | adaptive |
| PPP2R5C | ILMN_1789283 | T helper cells | adaptive |
| GOLGA8A | ILMN_1712469 | T helper cells | adaptive |
| C13orf34 | ILMN_1761486 | T helper cells | adaptive |
| LRBA | ILMN_1652160 | T helper cells | adaptive |
| BATF | ILMN_1668822 | T helper cells | adaptive |
| CD28 | ILMN_1749362 | T helper cells | adaptive |
| ITM2A | ILMN_2076600 | T-cells | adaptive |
| BCL11B | ILMN_1665761 | T-cells | adaptive |
| PRKCQ | ILMN_1733421 | T-cells | adaptive |
| GIMAP5 | ILMN_1769383 | T-cells | adaptive |
| CD6 | ILMN_1746565 | T-cells | adaptive |
| CD3E | ILMN_1739794 | T-cells | adaptive |
| CD2 | ILMN_1695025 | T-cells | adaptive |
| NCALD | ILMN_2343097 | T-cells | adaptive |
| CD3D | ILMN_2325837 | T-cells | adaptive |
| LCK | ILMN_2377109 | T-cells | adaptive |
| CD3G | ILMN_1717197 | T-cells | adaptive |
| TRAT1 | ILMN_1684943 | T-cells | adaptive |
| SKAP1 | ILMN_1751400 | T-cells | adaptive |
| CD96 | ILMN_2415786 | T-cells | adaptive |
| SH2D1A | ILMN_1705892 | T-cells | adaptive |
| NFATC3 | ILMN_2360028 | Tcm | adaptive |
| FOXP1 | ILMN_1738179 | Tcm | adaptive |
| DOCK9 | ILMN_1773413 | Tcm | adaptive |

| | | | |
|---|---|---|---|
| TRAF3IP3 | ILMN_1780397 | Tcm | adaptive |
| FYB | ILMN_1796537 | Tcm | adaptive |
| TIMM8A | ILMN_1722239 | Tcm | adaptive |
| AQP3 | ILMN_1651574 | Tcm | adaptive |
| RPP38 | ILMN_1680386 | Tcm | adaptive |
| NEFL | ILMN_1659086 | Tcm | adaptive |
| REPS1 | ILMN_2222768 | Tcm | adaptive |
| CASP8 | ILMN_1673757 | Tcm | adaptive |
| CEP68 | ILMN_1808500 | Tcm | adaptive |
| ATF7IP | ILMN_1716435 | Tcm | adaptive |
| USP9Y | ILMN_2056795 | Tcm | adaptive |
| CLUAP1 | ILMN_2242491 | Tcm | adaptive |
| POLR2J2 | ILMN_3241970 | Tcm | adaptive |
| HNRPH1 | ILMN_2101920 | Tcm | adaptive |
| MLL | ILMN_1668683 | Tcm | adaptive |
| KLF12 | ILMN_1762801 | Tcm | adaptive |
| PDXDC2 | ILMN_3251217 | Tcm | adaptive |
| PHC3 | ILMN_2179873 | Tcm | adaptive |
| CDC14A | ILMN_1655983 | Tcm | adaptive |
| PCM1 | ILMN_2042595 | Tcm | adaptive |
| INPP4B | ILMN_2198878 | Tcm | adaptive |
| CYorf15B | ILMN_1756506 | Tcm | adaptive |
| PSPC1 | ILMN_1724490 | Tcm | adaptive |
| CREBZF | ILMN_1784847 | Tcm | adaptive |
| SNRPN | ILMN_1656537 | Tcm | adaptive |
| CYLD | ILMN_2287941 | Tcm | adaptive |
| ST3GAL1 | ILMN_1683313 | Tcm | adaptive |
| ATM | ILMN_1779214 | Tcm | adaptive |
| PCNX | ILMN_1740010 | Tcm | adaptive |
| TXK | ILMN_1741143 | Tcm | adaptive |
| TBCD | ILMN_1795400 | Tem | adaptive |
| TBC1D5 | ILMN_1767433 | Tem | adaptive |
| VIL2 | ILMN_1795937 | Tem | adaptive |
| EWSR1 | ILMN_1697735 | Tem | adaptive |
| GDPD5 | ILMN_1701643 | Tem | adaptive |
| NFATC4 | ILMN_1796018 | Tem | adaptive |
| PRKY | ILMN_1772163 | Tem | adaptive |
| AKT3 | ILMN_2325610 | Tem | adaptive |
| LTK | ILMN_1679290 | Tem | adaptive |
| C7orf54 | ILMN_1655961 | Tem | adaptive |
| CCR2 | ILMN_2376431 | Tem | adaptive |
| FLI1 | ILMN_1665738 | Tem | adaptive |
| MEFV | ILMN_2115752 | Tem | adaptive |
| DDX17 | ILMN_1675124 | Tem | adaptive |
| ZNF764 | ILMN_1727938 | TFH | adaptive |
| C18orf1 | ILMN_2351795 | TFH | adaptive |

| | | | |
|---|---|---|---|
| SIRPG | ILMN_1771801 | TFH | adaptive |
| KCNK5 | ILMN_1766918 | TFH | adaptive |
| THADA | ILMN_1706818 | TFH | adaptive |
| PVALB | ILMN_2069224 | TFH | adaptive |
| POMT1 | ILMN_1743663 | TFH | adaptive |
| CXCL13 | ILMN_1718552 | TFH | adaptive |
| B3GAT1 | ILMN_1761093 | TFH | adaptive |
| SMAD1 | ILMN_1705392 | TFH | adaptive |
| CHGB | ILMN_1765966 | TFH | adaptive |
| PDCD1 | ILMN_1806725 | TFH | adaptive |
| SLC7A10 | ILMN_1681087 | TFH | adaptive |
| HEY1 | ILMN_1788203 | TFH | adaptive |
| TSHR | ILMN_1746712 | TFH | adaptive |
| MYO7A | ILMN_1811719 | TFH | adaptive |
| MYO6 | ILMN_1727080 | TFH | adaptive |
| MAF | ILMN_1719543 | TFH | adaptive |
| CHI3L2 | ILMN_2326273 | TFH | adaptive |
| PASK | ILMN_1667022 | TFH | adaptive |
| STK39 | ILMN_1791328 | TFH | adaptive |
| KIAA1324 | ILMN_1771482 | TFH | adaptive |
| MKL2 | ILMN_1690807 | TFH | adaptive |
| BLR1 | ILMN_1699669 | TFH | adaptive |
| PTPN13 | ILMN_2374683 | TFH | adaptive |
| ST8SIA1 | ILMN_2048011 | TFH | adaptive |
| TOX | ILMN_1788886 | TFH | adaptive |
| SH3TC1 | ILMN_1756595 | TFH | adaptive |
| ICA1 | ILMN_1814787 | TFH | adaptive |
| CD160 | ILMN_1742001 | Tgd | adaptive |
| C1orf61 | ILMN_1759652 | Tgd | adaptive |
| TARP | ILMN_1723944 | Tgd | adaptive |
| CSF2 | ILMN_1661861 | Th1 cells | adaptive |
| CMAH | ILMN_1704084 | Th1 cells | adaptive |
| HBEGF | ILMN_2121408 | Th1 cells | adaptive |
| DGKI | ILMN_1718266 | Th1 cells | adaptive |
| CTLA4 | ILMN_2261627 | Th1 cells | adaptive |
| GGT1 | ILMN_2368585 | Th1 cells | adaptive |
| LRP8 | ILMN_1677765 | Th1 cells | adaptive |
| APBB2 | ILMN_3251699 | Th1 cells | adaptive |
| CD38 | ILMN_2233783 | Th1 cells | adaptive |
| IL22 | ILMN_1735208 | Th1 cells | adaptive |
| LTA | ILMN_1795464 | Th1 cells | adaptive |
| EGFL6 | ILMN_2057479 | Th1 cells | adaptive |
| ATP9A | ILMN_2089073 | Th1 cells | adaptive |
| LRRN3 | ILMN_2048591 | Th1 cells | adaptive |
| CD70 | ILMN_1760247 | Th1 cells | adaptive |
| SYNGR3 | ILMN_1786379 | Th1 cells | adaptive |

| | | | |
|---|---|---|---|
| IL12RB2 | ILMN_1761921 | Th1 cells | adaptive |
| APOD | ILMN_1780170 | Th1 cells | adaptive |
| ZBTB32 | ILMN_1732981 | Th1 cells | adaptive |
| IFNG | ILMN_2207291 | Th1 cells | adaptive |
| DUSP5 | ILMN_1656501 | Th1 cells | adaptive |
| IL17RA | ILMN_1728724 | Th17 cells | adaptive |
| IL17A | ILMN_1774983 | Th17 cells | adaptive |
| RORC | ILMN_1734366 | Th17 cells | adaptive |
| MB | ILMN_1666109 | Th2 cells | adaptive |
| CDC25C | ILMN_1725260 | Th2 cells | adaptive |
| MICAL2 | ILMN_1785141 | Th2 cells | adaptive |
| ADCY1 | ILMN_2120555 | Th2 cells | adaptive |
| GATA3 | ILMN_2406656 | Th2 cells | adaptive |
| WDHD1 | ILMN_2377150 | Th2 cells | adaptive |
| PTGIS | ILMN_1667692 | Th2 cells | adaptive |
| CXCR6 | ILMN_1674640 | Th2 cells | adaptive |
| LAIR2 | ILMN_1807491 | Th2 cells | adaptive |
| AHI1 | ILMN_1791006 | Th2 cells | adaptive |
| CENPF | ILMN_1664516 | Th2 cells | adaptive |
| HELLS | ILMN_1783610 | Th2 cells | adaptive |
| NEIL3 | ILMN_1757697 | Th2 cells | adaptive |
| PHEX | ILMN_1668242 | Th2 cells | adaptive |
| ANK1 | ILMN_2317186 | Th2 cells | adaptive |
| IL26 | ILMN_2123182 | Th2 cells | adaptive |
| FOXP3 | ILMN_1768049 | TReg | adaptive |
| CCL1 | ILMN_2086965 | aDC | innate |
| LAMP3 | ILMN_2170813 | aDC | innate |
| INDO | ILMN_1656310 | aDC | innate |
| EBI3 | ILMN_1802653 | aDC | innate |
| CD209 | ILMN_1676372 | DC | innate |
| CCL13 | ILMN_1783593 | DC | innate |
| HSD11B1 | ILMN_1811370 | DC | innate |
| NPR1 | ILMN_1779897 | DC | innate |
| CCL17 | ILMN_1710186 | DC | innate |
| CCL22 | ILMN_2160476 | DC | innate |
| PPFIBP2 | ILMN_1675656 | DC | innate |
| TKTL1 | ILMN_1674009 | Eosinophils | innate |
| ACACB | ILMN_1763852 | Eosinophils | innate |
| IL5RA | ILMN_1756455 | Eosinophils | innate |
| CAT | ILMN_1651705 | Eosinophils | innate |
| EPN2 | ILMN_1815519 | Eosinophils | innate |
| THBS4 | ILMN_1736078 | Eosinophils | innate |
| SYNJ1 | ILMN_1701991 | Eosinophils | innate |
| THBS1 | ILMN_1686116 | Eosinophils | innate |
| IGSF2 | ILMN_1799278 | Eosinophils | innate |
| LRP5L | ILMN_1718633 | Eosinophils | innate |

| | | | |
|---|---|---|---|
| HRH4 | ILMN_1810424 | Eosinophils | innate |
| SMPD3 | ILMN_1802316 | Eosinophils | innate |
| RNASE2 | ILMN_1730628 | Eosinophils | innate |
| CYSLTR2 | ILMN_1773204 | Eosinophils | innate |
| RRP12 | ILMN_1767253 | Eosinophils | innate |
| RCOR3 | ILMN_1682095 | Eosinophils | innate |
| EMR1 | ILMN_1780601 | Eosinophils | innate |
| C9orf156 | ILMN_1700028 | Eosinophils | innate |
| KBTBD11 | ILMN_1784630 | Eosinophils | innate |
| KCNH2 | ILMN_1739987 | Eosinophils | innate |
| GALC | ILMN_1799744 | Eosinophils | innate |
| CLC | ILMN_1654875 | Eosinophils | innate |
| GPR44 | ILMN_1703326 | Eosinophils | innate |
| HES1 | ILMN_1710284 | Eosinophils | innate |
| CCR3 | ILMN_1763322 | Eosinophils | innate |
| FZD2 | ILMN_1653711 | iDC | innate |
| SYT17 | ILMN_1657760 | iDC | innate |
| SLC26A6 | ILMN_1785252 | iDC | innate |
| PPARG | ILMN_1800225 | iDC | innate |
| FABP4 | ILMN_1773006 | iDC | innate |
| CD1C | ILMN_1654210 | iDC | innate |
| TM7SF4 | ILMN_1793730 | iDC | innate |
| CSF1R | ILMN_1686623 | iDC | innate |
| HS3ST2 | ILMN_1712475 | iDC | innate |
| MS4A6A | ILMN_1797731 | iDC | innate |
| GUCA1A | ILMN_1662086 | iDC | innate |
| TACSTD2 | ILMN_1739001 | iDC | innate |
| CLEC10A | ILMN_2415303 | iDC | innate |
| CD1A | ILMN_1723520 | iDC | innate |
| ABCG2 | ILMN_1789641 | iDC | innate |
| GSTT1 | ILMN_1730054 | iDC | innate |
| CD1B | ILMN_1726230 | iDC | innate |
| VASH1 | ILMN_1796216 | iDC | innate |
| CARD9 | ILMN_1712532 | iDC | innate |
| CD1E | ILMN_2335754 | iDC | innate |
| MMP12 | ILMN_1768035 | iDC | innate |
| F13A1 | ILMN_1717163 | iDC | innate |
| CH25H | ILMN_1741021 | iDC | innate |
| GPC4 | ILMN_1789502 | Macrophages | innate |
| CD163 | ILMN_2379599 | Macrophages | innate |
| MSR1 | ILMN_1694400 | Macrophages | innate |
| CCL7 | ILMN_1683456 | Macrophages | innate |
| CXCL5 | ILMN_2171384 | Macrophages | innate |
| CHIT1 | ILMN_3249477 | Macrophages | innate |
| SULT1C2 | ILMN_2415329 | Macrophages | innate |
| BCAT1 | ILMN_1766169 | Macrophages | innate |

| | | | |
|---|---|---|---|
| COL8A2 | ILMN_1674050 | Macrophages | innate |
| GM2A | ILMN_2221046 | Macrophages | innate |
| COLEC12 | ILMN_1679049 | Macrophages | innate |
| DNASE2B | ILMN_1789130 | Macrophages | innate |
| MARCO | ILMN_1731503 | Macrophages | innate |
| ATG7 | ILMN_1790978 | Macrophages | innate |
| SCG5 | ILMN_2065773 | Macrophages | innate |
| KAL1 | ILMN_1750373 | Macrophages | innate |
| SGMS1 | ILMN_1740505 | Macrophages | innate |
| CHI3L1 | ILMN_3307868 | Macrophages | innate |
| CD84 | ILMN_1698367 | Macrophages | innate |
| CYBB | ILMN_1682312 | Macrophages | innate |
| MS4A4A | ILMN_2370336 | Macrophages | innate |
| CLEC5A | ILMN_1780465 | Macrophages | innate |
| CD68 | ILMN_2359907 | Macrophages | innate |
| ME1 | ILMN_1736042 | Macrophages | innate |
| FN1 | ILMN_2366463 | Macrophages | innate |
| PTGDS | ILMN_1664464 | Macrophages | innate |
| CEACAM8 | ILMN_1806056 | Mast cells | innate |
| SLC18A2 | ILMN_2070003 | Mast cells | innate |
| CTSG | ILMN_1680424 | Mast cells | innate |
| CMA1 | ILMN_1770772 | Mast cells | innate |
| SIGLEC6 | ILMN_2299095 | Mast cells | innate |
| TPSB2 | ILMN_3243238 | Mast cells | innate |
| HDC | ILMN_1792323 | Mast cells | innate |
| MAOB | ILMN_1727360 | Mast cells | innate |
| SLC24A3 | ILMN_1663519 | Mast cells | innate |
| NR0B1 | ILMN_1800160 | Mast cells | innate |
| GATA2 | ILMN_2102670 | Mast cells | innate |
| TAL1 | ILMN_1748450 | Mast cells | innate |
| ADCYAP1 | ILMN_1763344 | Mast cells | innate |
| CALB2 | ILMN_1748840 | Mast cells | innate |
| CPA3 | ILMN_1766551 | Mast cells | innate |
| TPSAB1 | ILMN_2169801 | Mast cells | innate |
| MPO | ILMN_1705183 | Mast cells | innate |
| SCG2 | ILMN_1703178 | Mast cells | innate |
| ABCC4 | ILMN_2194009 | Mast cells | innate |
| HPGD | ILMN_2166457 | Mast cells | innate |
| PRG2 | ILMN_1729314 | Mast cells | innate |
| MS4A2 | ILMN_1806721 | Mast cells | innate |
| LOC339524 | ILMN_1699320 | Mast cells | innate |
| PTGS1 | ILMN_1665100 | Mast cells | innate |
| SLC22A4 | ILMN_2050911 | Neutrophils | innate |
| CEACAM3 | ILMN_1743570 | Neutrophils | innate |
| MGAM | ILMN_1714643 | Neutrophils | innate |
| TNFRSF10C | ILMN_1672114 | Neutrophils | innate |

| | | | |
|---|---|---|---|
| SIGLEC5 | ILMN_1652381 | Neutrophils | innate |
| LILRB2 | ILMN_2312340 | Neutrophils | innate |
| PDE4B | ILMN_1782922 | Neutrophils | innate |
| ALPL | ILMN_1701603 | Neutrophils | innate |
| CD93 | ILMN_1704730 | Neutrophils | innate |
| IL8RB | ILMN_1680397 | Neutrophils | innate |
| HPSE | ILMN_2092850 | Neutrophils | innate |
| S100A12 | ILMN_1748915 | Neutrophils | innate |
| VNN3 | ILMN_1804935 | Neutrophils | innate |
| DYSF | ILMN_1810420 | Neutrophils | innate |
| HIST1H2BC | ILMN_1680937 | Neutrophils | innate |
| FPR1 | ILMN_2092118 | Neutrophils | innate |
| BST1 | ILMN_1770161 | Neutrophils | innate |
| G0S2 | ILMN_1691846 | Neutrophils | innate |
| CRISPLD2 | ILMN_1790689 | Neutrophils | innate |
| CREB5 | ILMN_1731714 | Neutrophils | innate |
| FCAR | ILMN_2279367 | Neutrophils | innate |
| KCNJ15 | ILMN_1675756 | Neutrophils | innate |
| CYP4F3 | ILMN_3251260 | Neutrophils | innate |
| SLC25A37 | ILMN_1715969 | Neutrophils | innate |
| FCGR3B | ILMN_2134453 | Neutrophils | innate |
| CSF3R | ILMN_2371280 | Neutrophils | innate |
| MADD | ILMN_1743583 | NK CD56bright cells | innate |
| XCL1 | ILMN_1718792 | NK CD56bright cells | innate |
| PLA2G6 | ILMN_1697654 | NK CD56bright cells | innate |
| MPPED1 | ILMN_1801778 | NK CD56bright cells | innate |
| FOXJ1 | ILMN_1802190 | NK CD56bright cells | innate |
| RRAD | ILMN_2186137 | NK CD56bright cells | innate |
| GTF3C1 | ILMN_1789839 | NK CD56dim cells | innate |
| GZMB | ILMN_2109489 | NK CD56dim cells | innate |
| KIR3DL2 | ILMN_2190842 | NK CD56dim cells | innate |
| KIR3DL1 | ILMN_2131828 | NK CD56dim cells | innate |
| KIR2DS1 | ILMN_2166812 | NK CD56dim cells | innate |
| SPON2 | ILMN_1676099 | NK CD56dim cells | innate |
| KIR2DL3 | ILMN_1667232 | NK CD56dim cells | innate |
| KIR3DL3 | ILMN_2082593 | NK CD56dim cells | innate |
| IL21R | ILMN_1661687 | NK CD56dim cells | innate |
| KIR3DS1 | ILMN_3242156 | NK CD56dim cells | innate |
| FLJ20699 | ILMN_1692464 | NK CD56dim cells | innate |
| KIR2DS5 | ILMN_1691803 | NK CD56dim cells | innate |
| TBXA2R | ILMN_1744251 | NK cells | innate |
| PSMD4 | ILMN_3307799 | NK cells | innate |
| TINAGL1 | ILMN_1807169 | NK cells | innate |
| FZR1 | ILMN_1753207 | NK cells | innate |
| ADARB1 | ILMN_1679797 | NK cells | innate |
| LDB3 | ILMN_2305599 | NK cells | innate |

| | | | |
|---|---|---|---|
| PDLIM4 | ILMN_1663976 | NK cells | innate |
| SLC30A5 | ILMN_1709728 | NK cells | innate |
| BCL2 | ILMN_1801119 | NK cells | innate |
| IGFBP5 | ILMN_1750324 | NK cells | innate |
| MRC2 | ILMN_1783946 | NK cells | innate |
| FUT5 | ILMN_1773310 | NK cells | innate |
| NCR1 | ILMN_1750761 | NK cells | innate |
| PRX | ILMN_1786648 | NK cells | innate |
| MCM3AP | ILMN_1784766 | NK cells | innate |
| SMEK1 | ILMN_1654998 | NK cells | innate |
| MAPRE3 | ILMN_1734290 | NK cells | innate |
| SPN | ILMN_1801040 | NK cells | innate |
| FGF18 | ILMN_1693483 | NK cells | innate |
| IL3RA | ILMN_1747344 | pDC | innate |

**Table S3. Association between the 6 CICs and Tumor infiltrating lymphocytes (TILs) measured by a single observer (S O'S) in pooled LMC dataset**

| TILs | CIC 1 | CIC 2 | CIC 3 | CIC 4 | CIC 5 | CIC 6 | P* | P** | P*** | P**** |
|---|---|---|---|---|---|---|---|---|---|---|
| Measured in whole tumor (%) | | | | | | | | | | |
| Brisk | 11.1 | 22.6 | 22.5 | 3.6 | 3.0 | 2.2 | $3x10^{-10}$ | $1.5x10^{-6}$ | $6x10^{-7}$ | $3.6x10^{-6}$ |
| Non-brisk | 85.7 | 73.7 | 76.1 | 67.7 | 82.1 | 88.9 | | | | |
| No TILs | 3.2 | 3.7 | 1.4 | 14.3 | 8.1 | 9.9 | | | | |
| Measured in tumor core sampled for mRNA extraction | | | | | | | | | | |
| Lots | 4.8 | 19.3 | 6.5 | 1.4 | 6.0 | 3.8 | $9x10^{-14}$ | $2.5x10^{-5}$ | $2.6x10^{-13}$ | 0.01 |
| Moderate | 12.9 | 21.8 | 14.3 | 8.8 | 23.8 | 10.1 | | | | |
| Some | 38.7 | 39.5 | 36.4 | 26.3 | 46.4 | 51.9 | | | | |
| None/Barely perceptible | 43.6 | 19.4 | 42.9 | 63.5 | 23.5 | 34.2 | | | | |

* Comparison of all 6 CICs; ** Comparison of all CICs except CIC 4; ***CIC 2 vs. CIC 4; **** CIC 3 vs CIC 4.

**Table S4. Prognostic effect of CICs in multivariable analysis including Breslow thickness and ulceration in the LMC**

| **Characteristic** | **CIC 4 vs. CIC 2** | | **CIC 4 vs. all others** | |
|---|---|---|---|---|
| | HR (95% CI) ^ | P | HR (95% CI) ^ | P |
| - | 3.0 (1.92, 4.53) | $8.1x10^{-7}$ | 2.5 (1.88, 3.29) | $1.8x10^{-10}$ |
| Age, sex, tumor site | 2.5 (1.59, 3.81) | $5.7x10^{-5}$ | 2.1 (1.58, 2.79) | $3.2x10^{-7}$ |
| Age, sex, site, mitotic rate, ulceration, Breslow thickness | 2.0 (1.19, 3.31) | 0.008 | 1.7 (1.25, 2.44) | 0.001 |
| Age, sex, site, mitotic rate, ulceration, Breslow thickness, vascular invasion | 1.9 (1.11, 3.19) | 0.02 | 1.6 (1.11, 2.39) | 0.01 |
| Age, sex, site, mitotic rate, ulceration, Breslow thickness, vascular invasion, mutation status $ | 1.7 (0.95, 3.04) | 0.08 | 1.6 (1.09, 2.28) | 0.02 |
| *CD8A*** | 3.6 (1.75, 7.37) | $5x10^{-4}$ | 2.5 (1.79, 3.44) | $5x10^{-8}$ |
| *CD8B*** | 2.3 (1.18, 4.36) | $8.1x10^{-7}$ | 2.2 (1.60, 2.97) | $8.2x10^{-7}$ |

^ Hazard ratio and 95% confidence interval for melanoma specific survival (MSS)

$ *BRAF-V600* and *NRAS* (codon 12, 13 or 61) mutation status. Although the difference between CIC 2 and CIC4 in this model with 10 variables is not significant (P=0.08), the hazard ration remains strong at 1.7.

** In these bivariate models, the CICs retain a significant prognostic value while neither *CD8A* nor *CD8B* are significant.

**Table S5. Consensus Immunome Cluster for paired primary/metastatic samples**

| Patient | Primary Immunome cluster | Metastasis Immunome cluster | Metastasis site & |
|---|---|---|---|
| 1 | High Immune CIC2 | Low Immune/ β-catenin High CIC4 | Nodal |
| 2 | High Immune CIC2 | High Immune CIC2* | Nodal |
| 3 | Intermediate Immune/Keratin Rich CIC5 | High Immune CIC2* | Nodal |
| 4 | Intermediate Immune/Keratin Rich CIC5 | Low Immune/β-catenin High CIC4 | Bone/Subcutaneous |
| 5 | Intermediate Immune/Keratin Poor CIC3 | Low Immune/β-catenin Low CIC1 | Nodal |
| 6 | Low Immune/Keratin Rich CIC6 | High Immune CIC2* | Nodal |
| 7 | Low Immune/β-catenin High CIC4 | Low Immune/β-catenin High CIC4 | Nodal |
| 8 | Low Immune/β-catenin High CIC4 | Intermediate Immune/Keratin Rich CIC5** | Subcutaneous |
| 9 | Low Immune/Keratin Rich CIC6 | Low Immune/Keratin Rich CIC6 | Skin/subcutaneous |

& No visceral metastases were available. Generally speaking the immune status of the metastases was poorer than the primary tumor (participant 1,4,5) or similar (participant 2, 7 and 9). In four metastases, the Immunome profile was suggestive of local tumor environment/contamination. In 3 nodal metastases (participant 2, 3 and 6) marked *, the immunome profile was suggestive of contamination of the tumor sample by nodal lymphocytes and in 1 marked ** there was evidence for a keratin signature in a skin metastasis.

**Table S6. Fold change between CIC2 (baseline) and CIC4 for β-catenin signaling components (P value from Mann-Whitney test)**

| Gene | LMC | | TCGA | |
|---|---|---|---|---|
| | **Fold change** | **P** | **Fold change** | **P** |
| *CTNNB1* | 1.59 | $4x10^{-15}$ | 1.26 | $6x10^{-4}$ |
| *SOX2* | 1.56 | 0.001 | 2.1 | 0.006 |
| *SOX11* | 1.84 | $3x10^{-7}$ | 2.1 | 0.001 |
| *c-MYC* | 1.61 | $2x10^{-12}$ | 1.6 | $3x10^{-8}$ |
| *TCF12* | 1.13 | 0.001 | 1.4 | $5x10^{-6}$ |
| *TCF1* | 1.03 | 0.38 | 1.1 | 0.02 |
| *APC2* | 1.70 | $4x10^{-6}$ | 2.1 | $2x10^{-7}$ |
| *VEGFA* | 1.36 | $5x10^{-6}$ | 1.8 | $10^{-8}$ |
| *APC* | 1.37 | $2x10^{-8}$ | 1.3 | $4x10^{-4}$ |
| *DKK2* | 0.57 | $3x10^{-10}$ | 0.54 | $3x10^{-4}$ |
| *DKK3* | 0.67 | $3x10^{-6}$ | 0.70 | 0.02 |
